# β7-Integrin worsens diet-induced metabolic syndrome in mice by altering intestinal lipid handling and colonic inflammatory monocyte infiltration

**DOI:** 10.1016/j.isci.2026.118042

**Published:** 2026-10-27

**Authors:** Sreepradha Eswaran, Agnes Seeger, Sabine Hamm, Kerstin Scheuer, Nicole Treichel, Juliane Hermann, Trong-Hieu Nguyen, Thomas Clavel, Joachim Jankowski, Norbert Wagner, Angela Schippers

**Affiliations:** 1 Department of Pediatrics, RWTH Aachen University, Aachen, Germany; 2 Functional Microbiome Research Group, Institute of Medical Microbiology, RWTH Aachen University, Aachen, Germany; 3 Institute for Molecular Cardiovascular Research (IMCAR), RWTH Aachen University, Aachen, Germany; 4 Institute of Molecular Medicine, RWTH Aachen University, Aachen, Germany

**Keywords:** β7 integrin, metabolic syndrome, innate immune cells, monocytes/macrophages, intestinal lipid metabolism

## Abstract

Western diet (WD)-induced metabolic syndrome (MetS) involves obesity, insulin resistance, dyslipidemia, and low-grade inflammation. While β7 integrin-expressing adaptive immune cells contribute to WD-induced MetS, the role of β7 integrin in innate immunity remains unclear. To address this, the effect of antibody-mediated β7 integrin blockade or β7 integrin deficiency on WD-induced MetS was studied in recombination activating gene-2-deficient mice. Loss or inhibition of β7 integrin protected mice from WD-induced obesity, dyslipidemia, and glucose intolerance, partially via loss of β7 integrin-MAdCAM-1 interactions. β7 integrin deficiency altered intestinal lipid handling and monocyte infiltration into the colon and adipose tissue. Adoptive transfer confirmed that β7-expressing monocytes promote metabolic dysfunction in WD-fed recipients. Phospho-signaling analyses revealed attenuated PI3K-Akt pathway activation in β7 integrin-deficient colonic macrophages, and antibody treatments caused minor shifts in fecal microbiota profiles. Thus, β7 integrin-expressing innate immune cells drive WD-induced metabolic inflammation through monocyte recruitment, macrophage activation, and altered intestinal fat metabolism.

## Highlights

• β7 integrin on innate immune cells drives metabolic syndrome
• β7 integrin alters intestinal lipid handling and monocyte infiltration
• β7 integrin upregulates PI3K-Akt signaling in macrophages
• β7 integrin contributes to insulin resistance

## Graphical abstract

**Figure:**
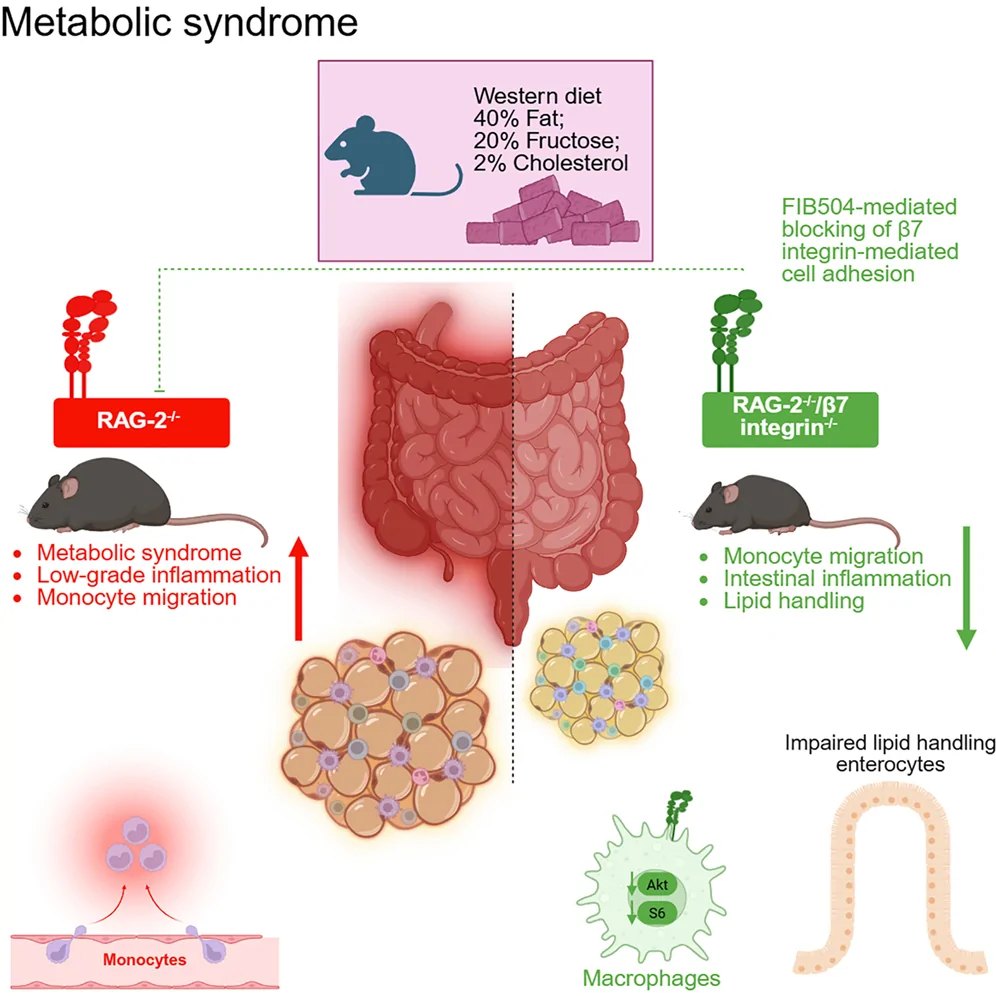

## Teaser

Immunology; Cell biology; Diet

## Introduction

Western diet (WD)-induced metabolic syndrome (MetS) with consequences like obesity, insulin resistance, dyslipidemia, metabolic dysfunction-associated steatotic liver disease (MASLD), hypertension, and increased cardiovascular risk is now regarded as a global public health challenge.^1^ The disease-promoting mechanisms are not completely understood. They include a complex interplay of signals interconnecting the gut, its microbiome, the liver, and adipose tissue, which cause a systemic low-grade inflammation thereby involving further tissues including the brain, pancreatic islets, blood vessels, and muscle.^2^^,^^3^ Immune cells from both innate and adaptive arms are central in mediating these inflammatory processes, acting through tissue infiltration, pro-inflammatory signaling, and dynamic interactions with the gut microbiota.^4^^,^^5^ As members of the innate immune system, the critical role of immigrated pro-inflammatory colonic macrophages, whose diet-induced activation can cause systemic metabolic disturbances like insulin resistance, is particularly noteworthy.^6^^,^^7^^,^^8^^,^^9^ With regard to the adaptive immune system, pathogenic IgG-producing B cells, IgA^+^ B cells, and CD4^+^ T, and CD8^+^ T cell-driven inflammation in adipose tissue play a pivotal role in obesity-associated inflammation and related diseases.^10^^,^^11^^,^^12^

Recruitment and immigration of immune cells are governed by the interaction of activated endothelia and epithelia with chemokine receptors and cell adhesion molecules expressed on circulating leukocytes. The adhesion molecule β7 integrin forms heterodimers with either the α4 or the αE (CD103) subunit and is expressed on all immune cells except neutrophils.^13^ While α4β7 integrin plays a key role in mediating immune cell homing to the intestine and mesenteric lymph nodes via binding to MAdCAM-1, αEβ7 integrin facilitates their retention in the intestinal epithelium by interaction with the epithelial ligand E-cadherin.^14^^,^^15^^,^^16^ β7 integrin has long been known to promote intestinal inflammation.^13^ In recent years, however, it has become clear that α4β7 integrin-expressing lymphocytes are also involved in metabolic dysregulation and liver diseases of different etiologies including MASLD.^17^^,^^18^^,^^19^

Inactivation of β7 integrin in the context of WD-induced obesity in mice, either by targeted mutagenesis or the blocking of antibodies, resulted in an improved clinical picture.^20^^,^^21^^,^^22^ This was expressed as better glucose tolerance and insulin sensitivity, reduced cellular infiltrates and crown-like structures in visceral adipose tissue and improved liver steatosis and atherosclerosis. These improvements were attributed mainly to decreased WD-induced recruitment and retention of CD4^+^ and CD8^+^ T cells to liver and intestine. However, β7 integrin is also involved in the intestinal localization of various innate immune cell subsets like conventional dendritic cells, plasmacytoid DCs and monocytes.^23^^,^^24^^,^^25^ with a potential pathogenic role in diet-induced metabolic dysregulation. By inactivating β7 integrin in the context of WD-treated recombination activating gene-2-deficient (RAG-2^-/-^) mice, we were able to demonstrate the contribution of β7 integrin-expressing innate immune cells, in particular monocytes/macrophages, to the development of diet-induced MetS.

## Results

### β7-integrin-expressing innate immune cells contribute to WD-induced MetS

To unravel the contribution of β7 integrin-expressing cells of the innate immune system to the development of obesity-associated MetS and to distinguish it from β7 integrin-mediated influences of the adaptive immune system, the model system of RAG-2-deficient mice,^26^ which lack mature B and T cells, was selected. Flow cytometric analysis of different CD45^+^ immune cell populations from the metabolic target organs of interest of RAG-2-deficient mice in homeostasis showed α4β7 integrin and αEβ7 integrin to be expressed on a considerable percentage of liver and gut cells, but there is hardly any α4β7 integrin expression in adipose tissue. As expected, the percentage of αEβ7 integrin-expressing leukocytes in the intraepithelial gut compartments is significantly higher than that of α4β7 integrin-expressing leukocytes (Figures S1A and S1B). Further characterization of myeloid cells showed that β7 integrin expression varies by cell type and tissue. Dendritic cells had the highest percentage of β7 integrin^+^ cells, especially in the colon lamina propria and mesenteric lymph nodes, which is presumably due to CD103^+^ (αE^+^ integrin) expression. Macrophages expressed β7 integrin less frequently, but those that did showed comparatively high mean fluorescence intensity in the colon, mesenteric lymph node and, epididymal white adipose tissue (EWAT) (Figures S1C–S1E).

MetS was induced by subjecting mice to a specific WD for 24 weeks. WD feeding of RAG-2-deficient mice resulted in an increase in body weight, EWAT to body weight ratio, and serum cholesterol that was comparable to similarly fed wild type (WT) mice. This demonstrates that MetS can develop without lymphocytes and underscores the applicability of this model to an investigation of metabolic disturbances driven by the innate immune system (Figures S2A–S2D). We next compared WD-fed RAG-2-deficient, RAG-2/β7-integrin double-deficient mice (RAG-2^-/-^/β7^-/-^), and RAG-2/MAdCAM-1 double-deficient (RAG-2^-/-^/MAdCAM-1^-/-^) mice. All metabolic parameters were significantly improved in RAG-2/β7-integrin double-deficient mice which, in contrast to the RAG-2-deficient mice, show less WD-induced gain in weight and EWAT/body weight ratio, no increase in serum cholesterol and serum triglycerides and no impaired glucose tolerance. This effect was accompanied by a reduced cumulative weekly food intake, which became noticeable after a treatment period of 4 weeks and decreased significantly by the end of the 24-week treatment period (Figures 1A–1I). In RAG-2-deficient mice with additional deficiency in the β7 integrin-ligand MAdCAM-1, most of the metabolic parameters were also significantly improved. We therefore concluded that loss of β7 integrin on innate immune cells protects from WD-induced obesity and glucose intolerance, at least partially via the β7 integrin-MAdCAM-1 interaction. A general impact of the additional mutations on the health status of the RAG-2-deficient mice under specific-pathogen-free conditions could be ruled out, as RAG-2/β7-integrin and RAG-2/MAdCAM-1 double-deficient mice did not undergo premature mortality and exhibited no differences in body weight, liver weight, serum parameters indicative of liver damage, or fat levels at one year of age (Figures S3A–S3G). The only notable observation was a lower EWAT weight in animals with an additional adhesion molecule deficiency. Moreover, chow-treated RAG-2-deficient and RAG-2/β7-integrin double-deficient mice exhibited no significant differences in insulin tolerance, fasted serum insulin, and serum insulin post-glucose gavage (Figures S4A–S4C).

**Figure 1:**
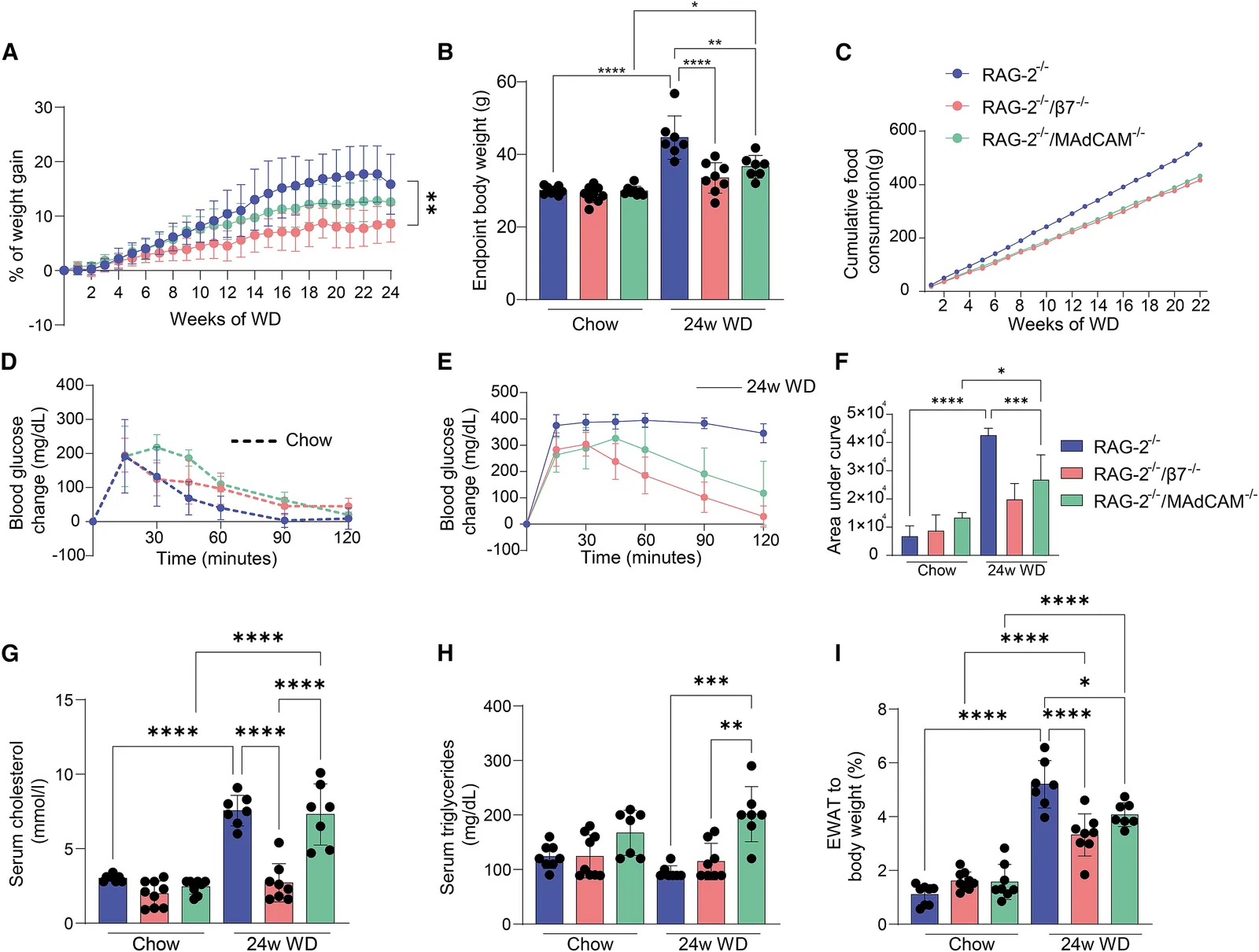
Loss of β7 integrin or MAdCAM-1 protects recombination activating gene-2-deficient (RAG-2^−/−^) mice from long-term WD-induced MetS RAG-2^−/−^ mice (shown in blue), RAG-2/β7 integrin double-deficient (RAG-2^−/−^/β7^−/−^) mice (shown in red), and RAG-2/MAdCAM-1 double-deficient (RAG-2^−/−^/MAdCAM-1^−/−^) mice (shown in green) were fed for 24 weeks with CD or WD. (A–C) Percentage of WD-induced body weight gain (RAG-2^−/−^ [*n* = 7], RAG-2^−/−^/β7^−/−^ [*n* = 8], and RAG-2^−/−^/MAdCAM-1^−/−^ [*n* = 7]), (B) endpoint body weight and (C) cumulative food intake (RAG-2^−/−^ [*n* = 8], RAG-2^−/−^/β7^−/−^ [*n* = 5], and RAG-2^−/−^/MAdCAM-1^−/−^ [*n* = 5]. A and C measured weekly over a 24-week course of WD treatment). (D) Endpoint glucose tolerance after CD-treatment (RAG-2^−/−^ [*n* = 5], RAG-2^−/−^/β7^−/−^ [*n* = 9], and RAG-2^−/−^/MAdCAM-1^−/−^ [*n* = 3]). (E and F) Endpoint glucose tolerance after WD-treatment (RAG-2^−/−^ [*n* = 7], RAG-2^−/−^/β7^−/−^ [*n* = 8], and RAG-2^−/−^/MAdCAM-1^−/−^ [*n* = 7]), and (F) corresponding quantification as area under curve. (G–I) Serum cholesterol, (H) serum triglycerides, and (I) percentage of EWAT to body weight from (RAG-2^−/−^) mice (CD [*n* = 8], WD [*n* = 7]), RAG-2^−/−^/β7^−/−^) mice (CD [*n* = 9], WD [*n* = 8]), and RAG-2^−/−^/MAdCAM-1^−/−^ mice (CD [*n* = 8], WD [*n* = 7]). Statistical significance was calculated either by the one-way ANOVA (A and F) and outliers were detected by ROUT method or two-way ANOVA and Tukey post-test (B and G–I). Values are represented as mean ± SD (A–E, and G–I) or mean ± SEM (F). ∗*p* ≤ 0.05, ∗∗ *p* ≤ 0.01, ∗∗∗ *p* ≤ 0.001, ∗∗∗∗ *p* ≤ 0.0001.

To exclude the possibility that the protective effect of β7 integrin-deficiency on metabolism is due to reduced food intake, a pair-feeding experiment was conducted in which RAG-2-deficient and RAG-2/β7-integrin double-deficient mice received only the smallest amount of food consumed by the RAG-2/β7-integrin double-deficient mice (3 g WD/day) over a period of 4 weeks. Interestingly, even with reduced food intake, RAG-2-deficient animals developed impaired glucose tolerance and showed higher serum cholesterol levels, suggesting that the observed difference in phenotype is independent of the ingested amount of WD (Figures S4D–S4F). To rule out any possible long-term effects of increased cumulative food intake all follow-up experiments were limited to 4-week diets with food *ad libitum*.

After 4 weeks of WD-feeding RAG-2-deficient and RAG-2/β7-integrin double-deficient mice showed similar body weight and food intake (Figures 2A and 2B); however, RAG-2/β7-integrin double-deficient mice displayed better glucose tolerance (Figures 2C and 2D), and lower serum cholesterol (Figure 2E). No significant differences in endpoint body weight were observed between mice fed chow diet (CD) and those fed WD (Figure 2F). EWAT to body weight ratio, however, was increased significantly by WD-feeding in RAG-2-deficient mice but not in RAG-2/β7-integrin double-deficient mice (Figure 2G).

**Figure 2:**
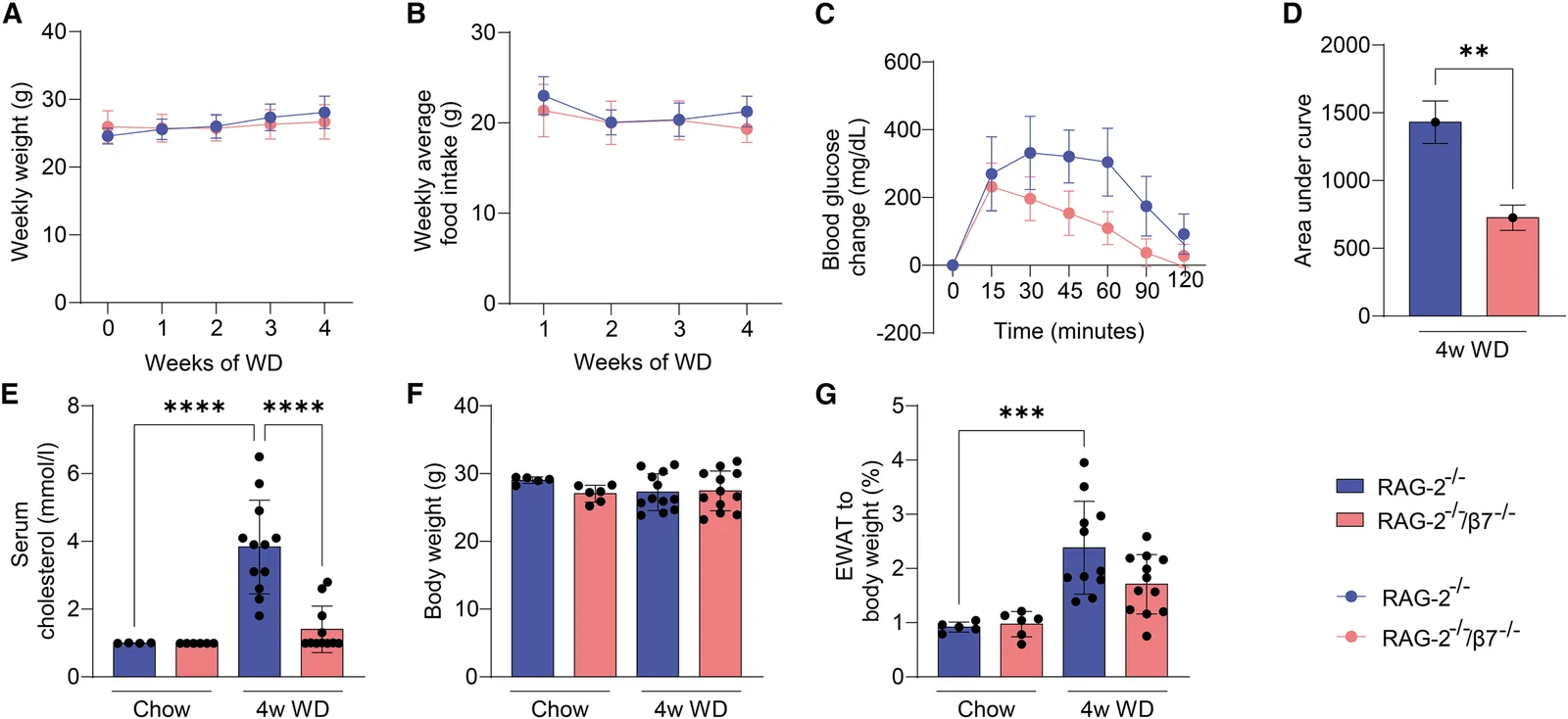
Loss of β7 integrin protects recombination activating gene-2-deficient (RAG-2^−/−^) mice from short-term WD-induced MetS RAG-2^−/−^ mice (shown in blue), and RAG-2/β7 integrin double-deficient (RAG-2^−/−^/β7^−/−^) mice (shown in red) were fed for 4 weeks with CD or WD. (A and B) Body weight (RAG-2^−/−^ [*n* = 12], RAG-2^−/−^/β7^−/−^ [*n* = 16]), and (B) weekly food intake (RAG-2^−/−^ [*n* = 14], RAG-2^−/−^/β7^−/−^ [*n* = 14]). (A and B measured weekly over a 4-week course of WD treatment). (C and D) Endpoint glucose tolerance after 4-week WD-treatment (RAG-2^−/−^ [*n* = 12], RAG-2^−/−^/β7^−/−^ [*n* = 16]) and (D) corresponding quantification as area under curve. (E–G) Serum cholesterol, (F) endpoint body weight, and (G) percentage of EWAT to body weight (RAG-2^−/−^ mice; CD [*n* = 4–5], WD [*n* = 12]), RAG-2^−/−^/β7^−/−^ mice (CD [*n* = 6], WD [*n* = 11–12]). Statistical significance was calculated either by *t*-test (D) or the one-way ANOVA (E–G) and Tukey post-test. Values are represented as mean ± SD (A–C, and E–G) or mean ± SEM (D). ∗*p* ≤ 0.05, ∗∗ *p* ≤ 0.01, ∗∗∗ *p* ≤ 0.001, ∗∗∗∗ *p* ≤ 0.0001.

### Loss of β7 integrin alters intestinal lipid handling in RAG-2-deficient mice

To learn more about the mechanisms that lead to the decreased net change in body mass of RAG-2/β7-integrin double-deficient mice, we performed a comparative metabolic analysis of CD-fed mice in PhenoMaster cages. RAG-2-deficient and RAG-2/β7-integrin double-deficient mice exhibited the expected diurnal variation in their respiratory exchange ratio (RER) with an increase in RER at night, when dietary nutrients such as carbohydrates, proteins and fats are used as the energy source after feeding (Figure 3A).

**Figure 3:**
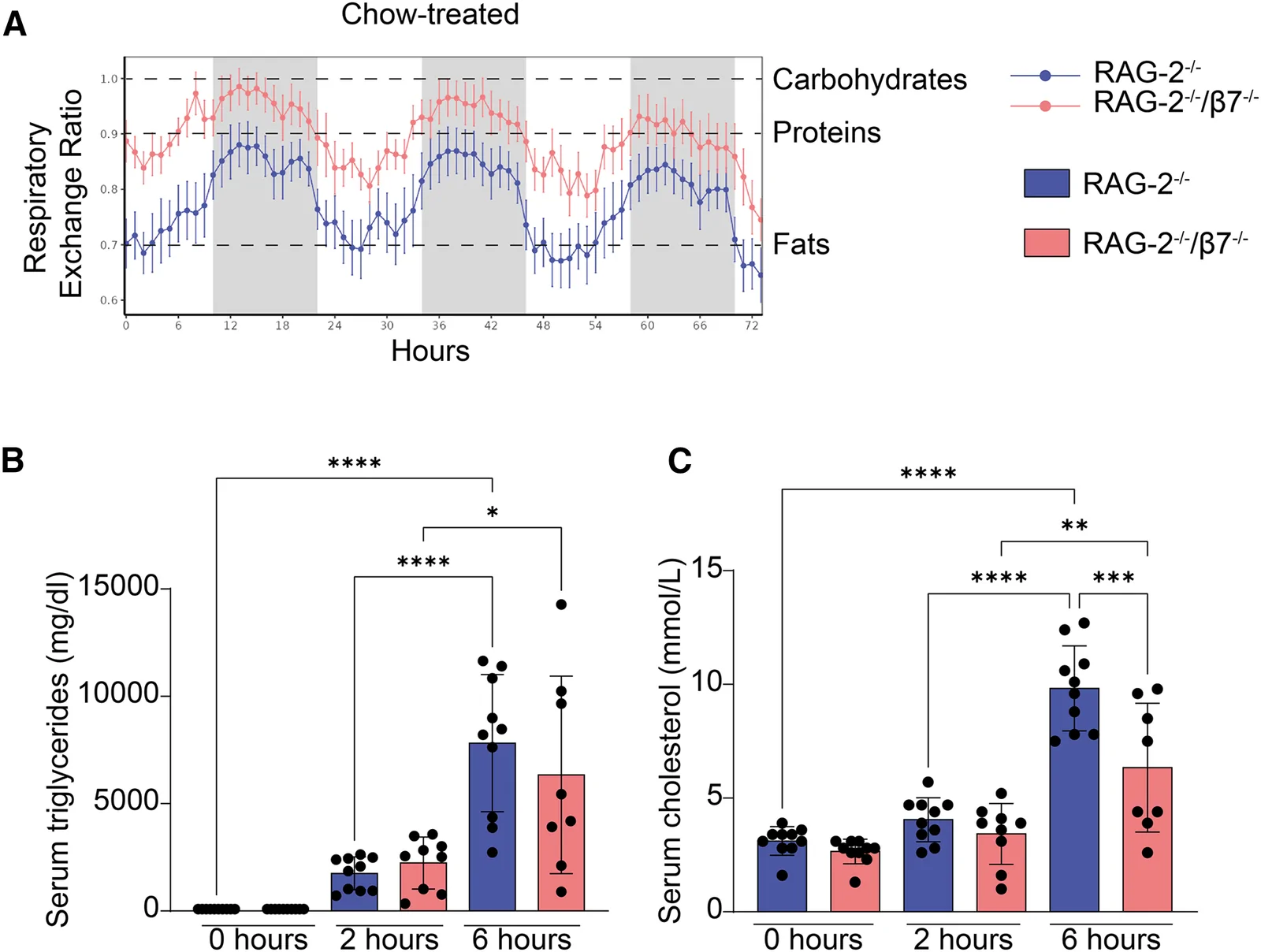
β7-integrin deficiency leads to an altered RER and altered intestinal lipid handling in chow-fed RAG-2-deficient (RAG-2^−/−^) mice (A) presents a comparison of chow-treated RAG-2^−/−^ mice (shown in blue, *n* = 8), and RAG-2/β7 integrin double-deficient (RAG-2^−/−^/β7^−/−^) mice (shown in red, *n* = 8) in metabolic cages. RER was calculated as the ratio of the volume of carbon dioxide exhaled to volume of oxygen inhaled. (B and C) Oral fat tolerance test on 12-week-old RAG-2^−/−^ mice (0 h [*n* = 10], 2 h [*n* = 10], 6 h [*n* = 10]), and RAG-2/β7 integrin double-deficient (RAG-2^−/−^/β7^−/−^) mice (0 h [*n* = 10], 2 h [*n* = 10], 6 h [*n* = 8]). Serum parameters were measured at the indicated time points following Poloxamer-407 administration and an olive oil gavage. (B) Serum triglycerides and (C) serum cholesterol. Values are represented as mean ± SD. Outliers were detected by ROUT method and statistical significance was calculated by two-way ANOVA test and Tukey post-test (B–C). ∗*p* ≤ 0.05, ∗∗ *p* ≤ 0.01, ∗∗∗ *p* ≤ 0.001, ∗∗∗∗ *p* ≤ 0.0001.

However, the comparatively higher RER of RAG-2/β7-integrin double-deficient mice indicates that they do not obtain their energy from fat but mainly from carbohydrates and proteins. This result was confirmed in a single experiment in which both groups of mice received WD, showing again the higher RER of RAG-2/β7-integrin double-deficient mice, which continues to oscillate in day and night cycles. In contrast, RAG-2-deficient mice display a flat RER across day and night cycles, pointing to their inability to use carbohydrates as an energy resource due to their WD-induced insulin resistance (Figure S4G).

Reduced utilization of fat as a food source in RAG-2/β7-integrin double-deficient mice may arise from impaired fat absorption. To determine, fecal lipid excretion, we quantified fecal triglycerides, cholesterol, and free fatty acids in feces from RAG-2- and RAG-2/β7 integrin double-deficient mice at baseline and after 4 weeks of WD (normalized per 100 mg dried feces). As expected, we observed a WD-induced increase in the excretion of lipids. However, no significant difference was found between any of the measured fecal lipids between RAG-2- and RAG-2/β7 integrin double-deficient mice at baseline and after 4 weeks of WD, indicating no overt intestinal malabsorption due to the β7 integrin deficiency (Figure S5). In contrast, an oral fat tolerance test following olive oil gavage revealed genotype-dependent differences in postprandial serum triglyceride dynamics. To determine intestinal fat absorption, mice that were starved for 6 hours, received an i.p. administration of Poloxamer 407 to block triglyceride clearance, directly before an oral gavage of olive oil. Serum triglycerides and cholesterol were measured directly, after 2 hours and after 6 hours. Directly after gavage, there was no difference in serum triglyceride and cholesterol levels between the two mouse groups. However, the increase in serum triglycerides and serum cholesterol was significantly attenuated in RAG-2/β7-integrin double-deficient mice compared to RAG-2-deficient mice, indicating altered intestinal lipid handling in the absence of β7 integrin (Figures 3B and 3C). While these findings are consistent with the hypothesis that altered intestinal lipid handling may contribute to protection against WD-induced MetS, the current data remain correlative. Additional measurements, such as fecal caloric loss, or direct intestinal triglyceride absorption assays, would be required to establish a causal relationship, but were beyond the scope of this study.

### Amelioration of WD-induced metabolic syndrome in lymphopenic β7 integrin-deficient mice is accompanied by a diminished influx of intestinal monocytes-macrophages

A previously reported obesity-associated accumulation of pro-inflammatory macrophages within the colon and adipose tissue was thought to result from increased circulating Ly6C^high^ monocytes and possibly to contribute to obesity-associated low-grade inflammation and MetS.^8^^,^^27^ In comparison to RAG-2-deficient mice, EWAT of RAG-2/β7-integrin double-deficient mice, fed for 24 weeks with WD, was characterized by significantly lower macrophage numbers and fewer CD68^+^ crown-like structures, indicating a lower degree of inflammation (Figures S6A and S6B).

In addition, although not significant, matrix-assisted laser desorption ionization mass spectrometric imaging (MALDI-MSI) analysis showed a WD-induced increase in the colonic macrophage marker FCGR1 in RAG-2-deficient but not in RAG-2/β7-integrin double-deficient mice (Figure S6C), indicating impaired migration of β7 integrin-deficient monocytes into the colon.

To determine whether there are any differences between RAG-2-deficient and RAG-2/β7-integrin double-deficient mice in the diet-induced recruitment of monocytes/macrophages into colonic adipose, and small intestinal tissues, further flow cytometric analyses were performed after 4 weeks of WD feeding. WD treatment resulted in a significant increase in “incoming monocytes” (CD45^+^CD11b^+^Ly6C^+^) in the colonic lamina propria and adipose tissue of RAG-2-deficient mice, which was not seen in the double-deficient mouse mutant (Figures 4A and 4B). This result was supported by the comparatively lower expression of *Ccl2*, *Cd68*, *and Adgre1* (F4/80) in colonic tissue extracts of WD-treated RAG-2/β7-integrin double-deficient mice (Figure 4C). Moreover, deficiency of β7 Integrin in WD-treated RAG-2-deficient mice was associated with decreased frequencies of small intestinal macrophages (CD45^+^CD11b^+^CD64^+^) (Figure 4D). According to the literature, cell populations expressing these markers should exhibit a pro-inflammatory phenotype.^28^^,^^29^

**Figure 4:**
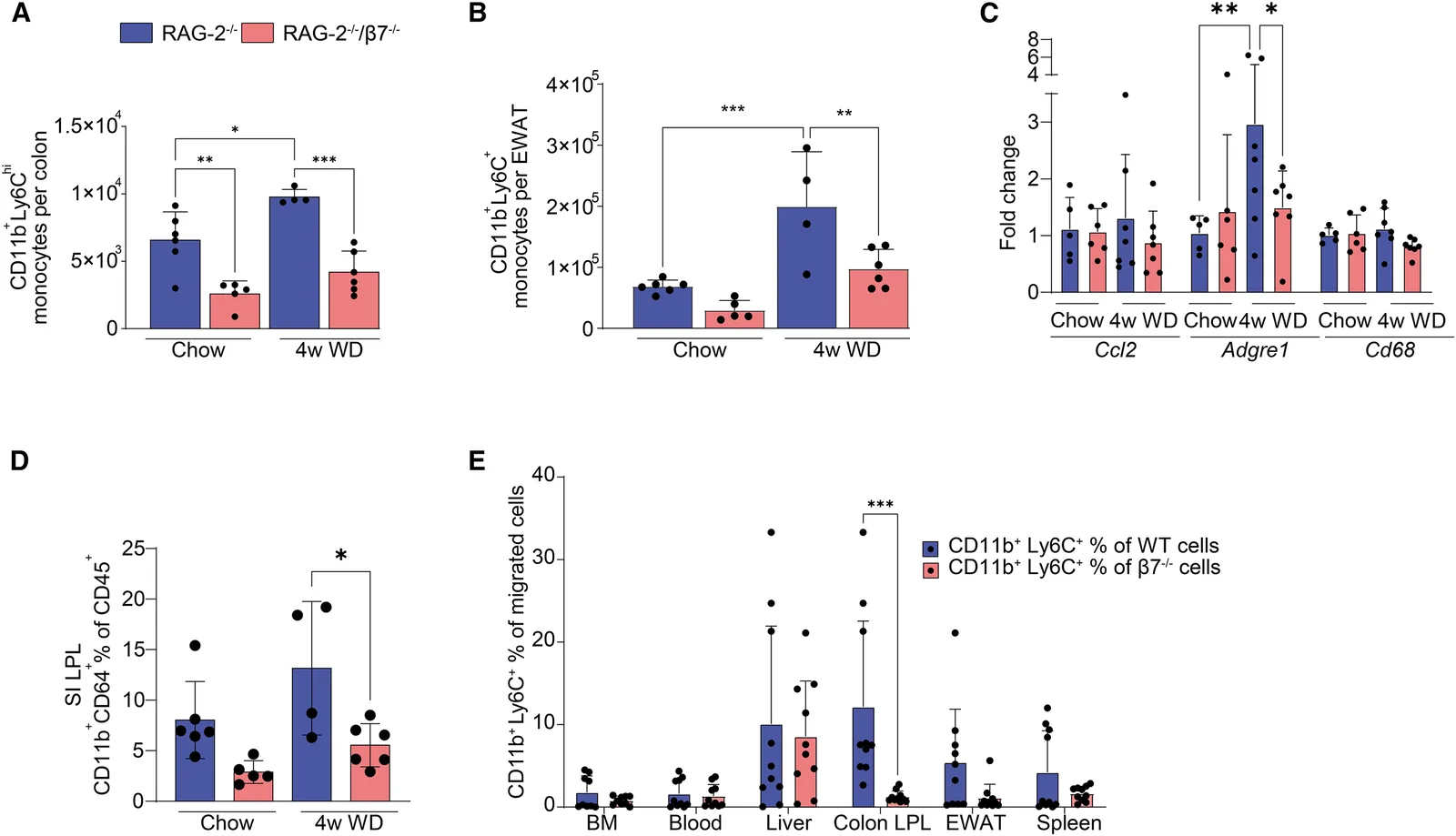
Ameliorated MetS correlates with decreased monocyte immigration and macrophage numbers in lymphopenic β7 integrin-deficient mice RAG-2-deficient (RAG-2^−/−^) mice (shown in blue) and RAG-2/β7 integrin double-deficient (RAG-2^−/−^/β7^−/−^) mice (shown in red) were fed for 4 weeks with CD or WD. (A, B, and D) Quantification of monocytes or macrophages by flow cytometry after 4 weeks of CD or WD treatment in RAG-2^−/−^ (CD-fed, [*n* = 6], WD-fed, [*n* = 4] and RAG-2^−/−^/β7^−/−^) (CD-fed, [*n* = 5], WD-fed, [*n* = 6] mice). (A) Monocyte numbers (CD45^+^ CD11b^+^ Ly6C^high^) in the colonic lamina propria and (B) monocyte numbers (CD45^+^ CD11b^+^ Ly6C^+^) in EWAT. (C) mRNA levels of *Ccl2*, *Adgre1* (F4/80), and *Cd68* in colonic tissue homogenates after 4-week WD treatment of RAG-2^−/−^ (WD-fed, *n* = 7) and RAG-2^−/−^/β7^−/−^ (WD-fed, *n* = 7) mice. For quantification, values are expressed as fold increase over the mean values obtained for colon tissue from CD-fed mice of the respective genotype. (D) Percentage of macrophages (CD11b^+^ CD64^+^) in the small intestinal lamina propria represented as frequency of CD45^+^ cells. (E) Comparison of proportions of immigrated monocytes (CD11b^+^ Ly6C^+^) 16 hours after adoptive transfer of differently stained BM cells from WT and β7^−/−^ donors into 4 weeks WD-treated RAG-2^−/−^ (*n* = 10) recipient mice. Gating strategies for flow cytometric data can be found in the supplemental information. Statistical significance was calculated by one-way ANOVA (A–C and E) or two-way ANOVA test (D). Values are represented as mean ± SD. ∗*p* ≤ 0.05, ∗∗ *p* ≤ 0.01, ∗∗∗ *p* ≤ 0.001, ∗∗∗∗ *p* ≤ 0.0001.

To determine the impact of β7 integrin on obesity-induced migration of monocytes from the peripheral blood into the colon, we intravenously injected equal numbers of differently stained bone marrow (BM) cells from β7 integrin-deficient and WT mice into RAG-2-deficient mice that had previously been fed WD for 4 weeks. At 16 h after transfer we monitored the frequencies of CD11b^+^Ly6C^+^ cells that had arrived in different organs. There was no difference in the percentage of these cells arriving in BM, liver, spleen, and remaining in blood, between WT and β7 integrin-deficient donors. However, in the colonic lamina propria, we detected a significantly lower percentage of these presumably pro-inflammatory cells from β7 integrin-deficient donors compared to WT donors, with a trend toward reduction in the EWAT (Figure 4E).

Results from experiments where RAG-2-deficient mice were subjected to 4 weeks of WD with concurrent i.p. administration of the anti-β7 integrin antibody FIB 504 or the corresponding isotype control are in line with this (Figure 5). Although no effect on glucose tolerance was observed (Figures S7A and S7B), FIB 504 injections resulted in improved insulin sensitivity (Figures 5B and 5C), which was accompanied by lower Ly6C^+^ monocyte frequencies in intestinal and adipose tissues (Figures 5D–5J). We therefore concluded that loss of β7 integrin disrupts diet-induced migration of macrophage precursors into the colon, small intestine and adipose tissue. Interestingly, we also observed a reduced expression of *Carboxylesterase 1d* (*Ces1d*) and other genes involved in regulation of cholesterol, fatty acid and triglyceride homeostasis in tissue homogenates of small intestines from FIB 504 treated mice (Figure 5K), suggesting a change in the lipid metabolism of enterocytes.

**Figure 5:**
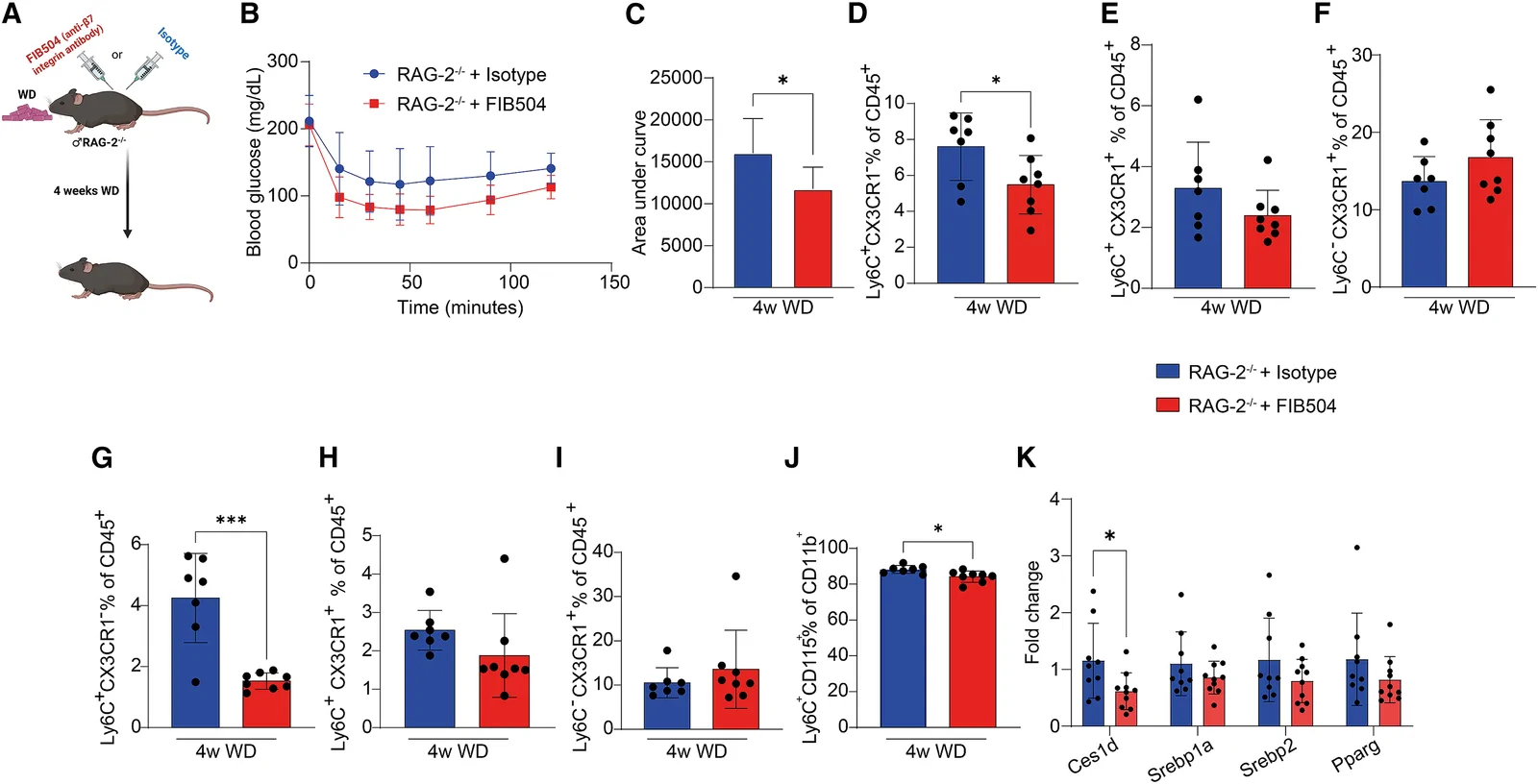
Antibody-mediated blocking of β7 integrin improves insulin sensitivity and correlates with decreased monocyte immigration in lymphopenic mice (A) Schematic overview of experimental design. RAG-2^−/−^ mice were fed for 4 weeks with a WD and given an IgG isotype antibody (shown in blue, (n = 7-9)) or FIB 504 monoclonal antibody against β7 integrin (shown in red, [*n* = 8–10]). (B and C) Endpoint insulin tolerance test and (C) corresponding quantification as area under curve. (D–F) Flow cytometric analysis of colonic monocyte populations (Ly6C^+^ CX3CR1^-^, Ly6C^+^ CX3CR1^+^, and Ly6C^-^ CX3CR1^+^) and (G–I) small intestinal monocyte populations (Ly6C^+^ CX3CR1^-^ , Ly6C^+^ CX3CR1^+^, and Ly6C^-^ CX3CR1^+^) represented as frequency of CD45^+^ cells. (J) Ly6C^+^ CD115^+^ monocyte frequency of CD11b^+^ cells in EWAT. (K) mRNA levels of *Carboxylesterase 1D* (*Ces1d*), *Sterol regulatory element-binding protein 1a* (*Srebp1a*), *Sterol regulatory element binding transcription factor 2* (*Srebp2*), and *Peroxisome proliferator-activated receptor gamma* (*Pparg*) in small intestine tissue homogenates. Gating strategies for flow cytometric data can be found in the supplemental information. Statistical significance was calculated by *t* test and values are represented as mean ± SD (B and D–K); mean ± SEM (C). ∗*p* ≤ 0.05, ∗∗∗*p* ≤ 0.001 .

### Antibody-mediated blocking of β7 integrin in WD-treated lymphopenic mice has little effect on the fecal microbiota composition

Colonic macrophages may influence and are influenced by the gut microbiota, and WD-induced alterations in this network may cause alterations in gut barrier function, metabolic inflammation and insulin resistance.^30^^,^^31^ For this reason, we also compared WD-induced changes in the fecal microbiota of RAG-2-deficient mice treated with the anti-β7 integrin antibody FIB 504 or the corresponding isotype by 16S rRNA gene amplicon sequencing. RAG-2-deficient mice originating from five litters were randomly assigned to control or treatment groups, placed in single-cages and fecal samples were collected before and at the end of the 4-week treatment. Sequencing depth was sufficient (Figure S8), resulting in 12,386 ± 4811 high-quality, merged reads per sample, representing a total of 202 Operational Taxonomic Units (OTUs), with 82 ± 30 per sample. Differences in fecal microbiota profiles between the antibody-treated groups before and after 4 weeks of WD feeding were assessed by non-metric multidimensional scaling (NMDS) of generalized UniFrac distances, which revealed a marked alteration due to WD feeding but no distinct difference in profiles between the two antibody-treated groups (Figure 6A). In addition, a significant difference in α-diversity was only observed due to WD feeding (Figures 6B and 6C). WD feeding triggered changes in the relative abundance of major bacterial phyla (Figure 6D): *Bacillota* increased sharply, while *Bacteroidota* were reduced. A WD-induced increase in the relative abundance of *Bacillota* was mainly due to *Enterococcaceae*, *Erysipelotrichaceae*, Streptococcaceae, and *Oscillospiraceae*, while, *Rikenellaceae*, *Muribaculaceae*, and *Prevotellaceae* contributed to the reduction of *Bacteroidota* (Figure 6E). Interestingly, at the family level, a comparatively higher relative abundance of *Erysipelotrichaceae* was detectable in feces of WD-fed animals treated with control antibody, while animals receiving the anti-β7 integrin antibody showed a significantly higher relative abundance of *Lachnospiraceae*.

**Figure 6:**
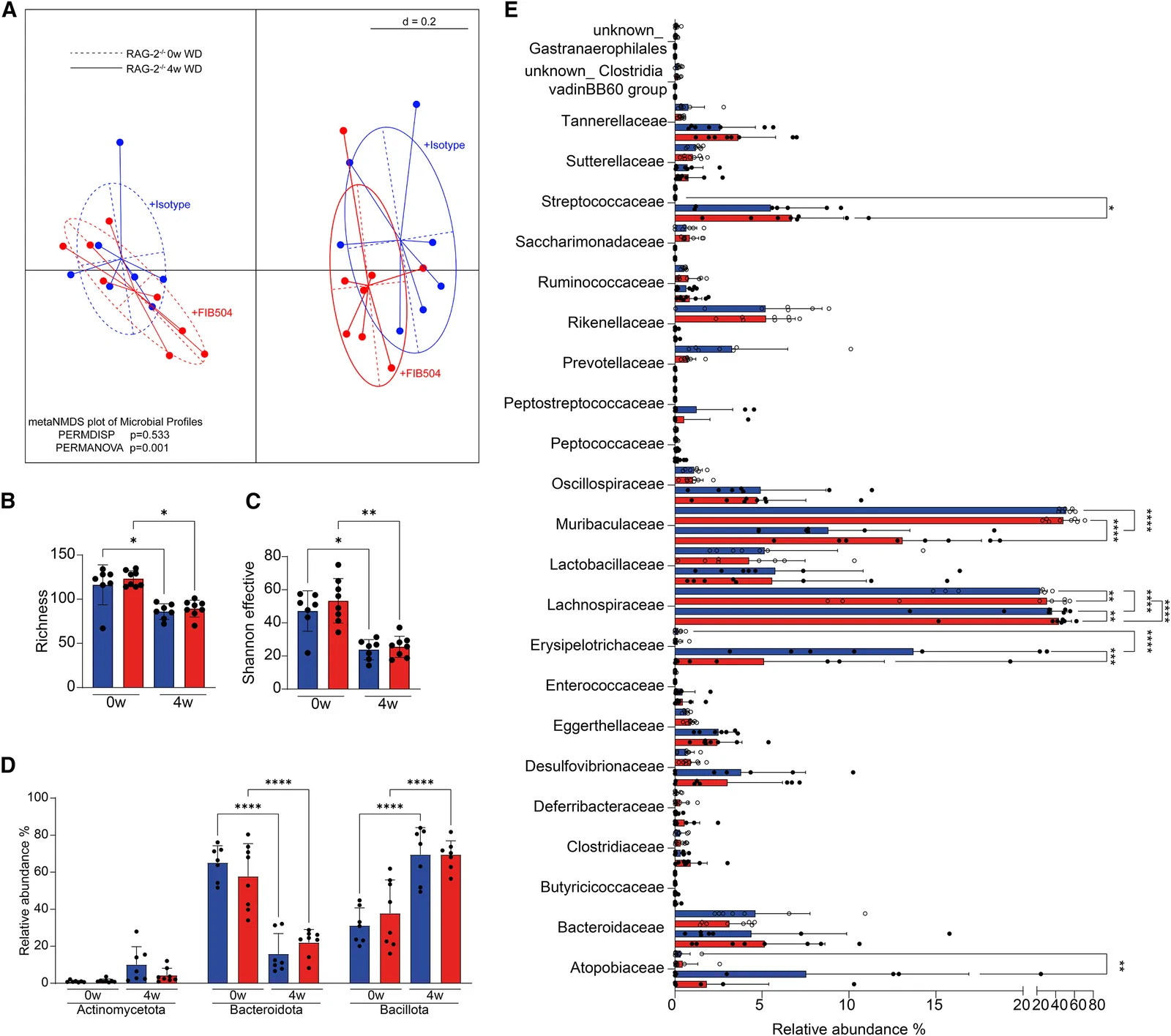
Antibody-mediated blocking of β7 integrin has only minor effects on the fecal microbiota composition of WD-treated lymphopenic mice Fecal samples were collected from RAG-2-deficient (RAG-2^−/−^) mice treated with the anti-β7 integrin antibody FIB 504 (shown in red, [*n* = 8]) or the corresponding isotype (shown in blue, [*n* = 7]) before 0 weeks (0 w) and after 4 weeks (4 w) of WD feeding. The diversity of OTUs within a given sample (alpha-diversity) and between samples (beta-diversity) was calculated in R using Rhea. (A) Beta-diversity of experimental groups shown as meta NMDS plot of microbiota profiles based on generalized UniFrac distances from mice at the indicated time points. Individual time points that were considered for calculations have been marked within their respective circles. (B and C) Alpha-diversity (richness), and (C) Shannon effective of experimental groups shown as number of observed OTUs. (D and E) Relative abundance of selected phyla (D), and families (E). Statistical significance was calculated by (A) PERMANOVA, (B and C) one-way ANOVA with Dunn’s post-test, and (D and E) two-way ANOVA. Values are represented as mean ± SD (B–E). ∗ *p* ≤ 0.05, ∗∗ *p* ≤ 0.01, ∗∗∗*p* ≤ 0.001, ∗∗∗∗ *p* ≤ 0.0001.

### β7-Integrin-expressing pro-inflammatory monocytes promote the development of metabolic syndrome

Next, we wanted to determine whether β7 integrin-expressing monocytes contribute to the WD-induced MetS. To this end, we administered CD115-enriched BM cells from WT or β7 integrin-deficient mice intravenously into CCR2-deficient mice at the start and half-way through four weeks of WD treatment. Due to a dysfunctional egress of monocytes from the BM, CCR-2-deficient mice have fewer of these cells at sites of inflammation.^32^ Since they have also been shown to be less affected by MetS, they represent an ideal model to compare the function of different monocyte populations by means of adoptive transfer experiments. No significant differences in food consumption were observed between the experimental groups. Transfer of CD115^+^ WT monocytes resulted in increased body weight, EWAT weight, serum triglycerides, fasted blood glucose, and serum MCP-1 levels, which was not seen after transfer of monocytes from β7 integrin-deficient mice (Figures 7A–7E). Moreover, a higher percentage of CD11b^+^CD115^+^Ly6C^+^ pro-inflammatory monocytes was detected in colon and EWAT of WD-fed CCR2-deficient mice after transfer of CD115^+^ WT donor cells than was measured in β7 integrin-deficient donors (Figure 7F). Furthermore, as was found for RAG-2-deficient mice, adoptive transfer of differently stained BM cells from β7 integrin-deficient and WT mice into WD-fed CCR-2-deficient mice showed a reduced capacity of β7 integrin-deficient CD11b^+^ CD115^+^ Ly6G^-^ monocytes to migrate into colon and adipose tissue (Figure 7G). We therefore conclude that β7 integrin-expressing monocytes migrating into the colon and adipose tissue contribute to the development of WD-induced MetS. Further studies, including metabolic cage analysis to assess energy expenditure and substrate utilization, will be required to determine the contribution of these parameters to the β7 integrin-dependent metabolic phenotype.

**Figure 7:**
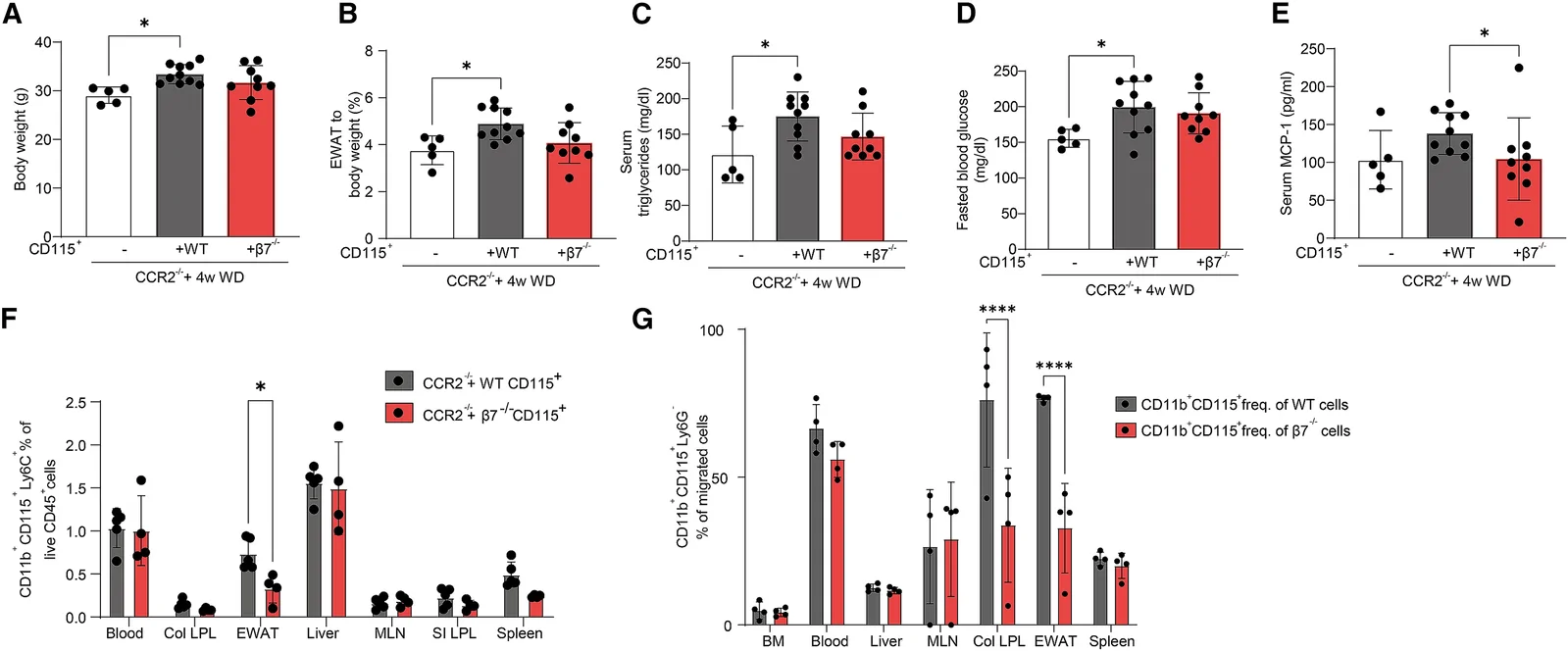
β7 integrin-mediated immigration of monocytes contributes to WD-induced MetS (A–F) C-C chemokine receptor-2-deficient (CCR-2^−/−^) mice were adoptively transferred with CD115^+^ BM monocytes from wild type (WT) mice (shown in gray, [*n* = 5–10] or β7 integrin-deficient [β7^−/−^] mice; shown in red, [*n* = 4–9]) or not transferred (shown in white, [*n* = 5]), fed for 4-week with WD and assessed for development of MetS. (A) Endpoint body weight, (B) EWAT/body weight percentage, (C) serum triglycerides, (D) serum fasted blood glucose, (E) serum monocyte chemoattractant protein-1 (MCP-1), and (F) flow cytometric analysis of monocytes (Live CD45^+^ CD11b^+^ Ly6C^+^ CD115^+^) in the indicated organs. (G) Comparison of the migration efficiency of BM monocytes (CD11b^+^ CD115^+^ Ly6G^-^) from WT and β7^−/−^ donors into the indicated organs of 4-week WD-treated CCR-2^-/-^ recipient mice (*n* = 4) 16 h after intravenous transfer by flow cytometric quantification. Gating strategies for flow cytometric data can be found in the supplemental information. Outliers were detected by ROUT method and statistical significance was calculated by one-way ANOVA test (A–D), *t* test (E) or two-way ANOVA test (F and G). Values are represented as mean ± SD (A–G). ∗*p* ≤ 0.05, ∗∗∗∗ *p* ≤ 0.0001.

### Loss of β7 integrin alters responses of intestinal macrophages in WD-treated RAG-2 deficient mice

Next, we wanted to address the question whether absence of β7-integrin causes changes in intestinal macrophage function that might contribute to the observed amelioration of WD-induced MetS in RAG-2/β7-integrin double-deficient mice. For this purpose, we compared the gene expression of FACS sorted macrophages (CD45^+^ SiglecF^-^ Ly6G^-^ CD11b^+^ CD64^+^) from the colon (Figure 8B) or small intestine (Figure 8C) of 4-week WD fed male RAG-2-deficient and RAG-2/β7-integrin double-deficient mice by bulk RNA-seq analysis. Our criterion for a significant change in gene expression is FDR < 0.05 and absolute log fold change ≥ 1. 182 genes were significantly upregulated in the colon of RAG-2-deficient mice compared to RAG-2/β7-integrin double-deficient mice, whereas 12 genes showed significantly higher expression in RAG-2/β7-integrin double-deficient mice (Figure 8B). In the small intestine of RAG-2-deficient mice only one gene, *Reg3g*, was identified as significantly upregulated, while four genes showed increased expression in double-deficient mice (Figure 8C).

**Figure 8:**
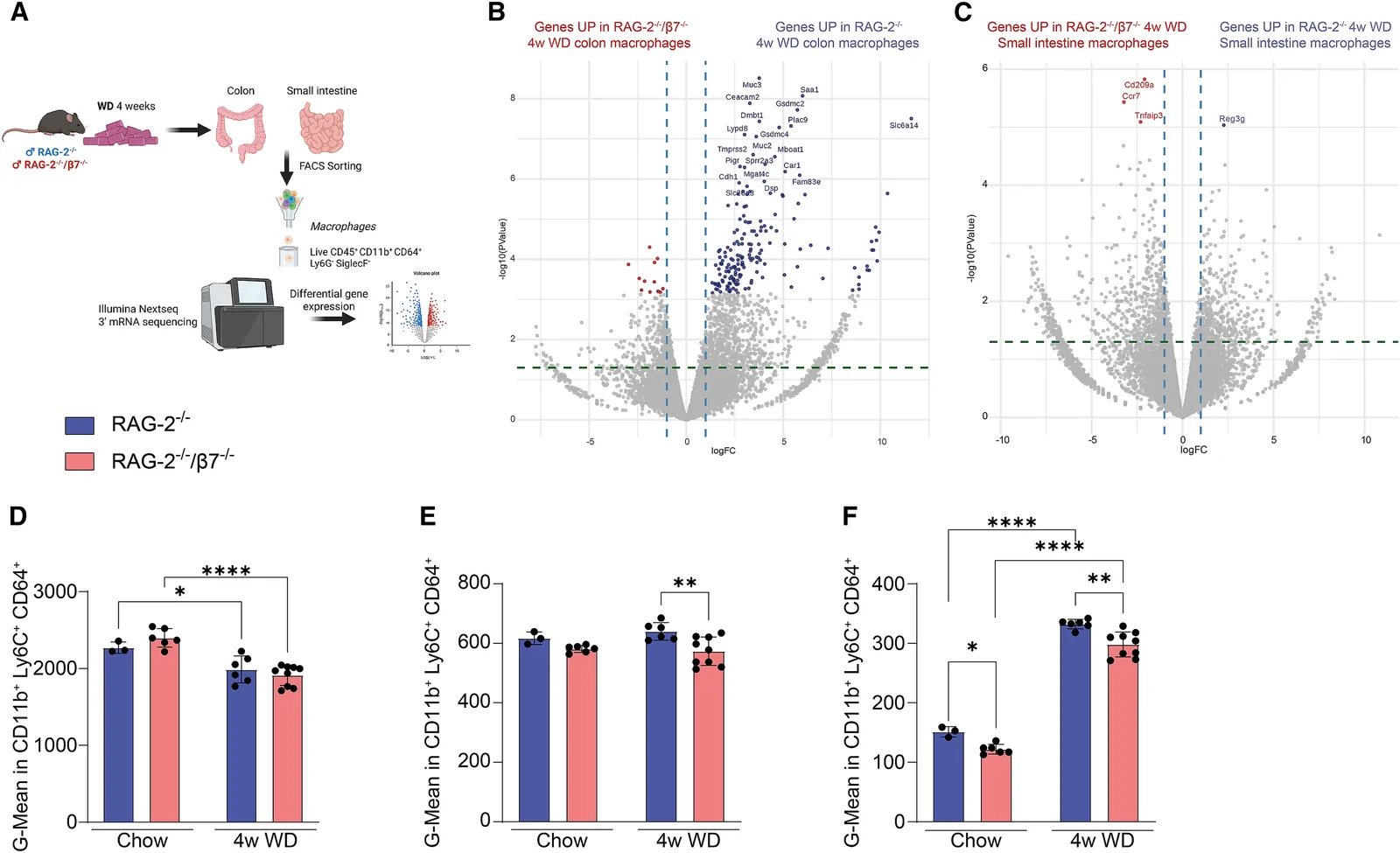
Loss of β7 integrin alters intestinal macrophage response to WD (A) Schematic representation depicting isolation of FACS-sorted colonic and small intestinal macrophages (CD45^+^ SiglecF^-^ Ly6G^-^ CD11b^+^ CD64^+^) from 4-week WD-fed RAG-2-deficient (RAG-2^−/−^) and RAG-2/β7-integrin double-deficient (RAG-2^−/−^/β7^−/−^) mice for bulk RNA-seq. (B and C) Volcano plots depicting differentially expressed genes between macrophages of WD-fed RAG-2^−/−^ and RAG-2^−/−^/β7^−/−^ mice in (B) colon lamina propria and (C) small intestine lamina propria. Genes with an FDR < 0.05 are highlighted, upregulated genes in RAG-2-deficient macrophages are shown in blue (right) and upregulated genes in RAG-2/β7-integrin double-deficient macrophage populations in red (left). (D–F) Analysis of mTOR/Akt signaling pathway activation and ribosomal protein S6 in monocytes-macrophages (CD11b^+^ CD64^+^ Ly6C^+^) from the colonic lamina propria of a 4-week WD or CD treated RAG-2^−/−^ (CD-fed: *n* = 3, WD-fed: *n* = 6) and RAG-2^−/−^/β7^−/−^ (CD-fed: *n* = 6, WD-fed: *n* = 9) mice by flow cytometry. Data are shown as Geometric mean fluorescence intensity (G-Mean) of (D) phosphorylated mechanistic target of rapamycin (mTOR), (E) protein kinase B (AKT), and (F) ribosomal protein S6 (S6). Gating strategies for flow cytometric data can be found in the supplemental information. Data from two independent experiments were pooled, with each experiment consisting of cells pooled from 5 to 6 mice (B and C). Values are represented as mean ± SD (D–F). Statistical significance was calculated by Benjamini-Hochberg adjusted *p* value (B and C), two-way ANOVA test (D–F) and Tukey’s post-test. ∗*p* ≤ 0.05, ∗∗ *p* ≤ 0.01, ∗∗∗ *p* ≤ 0.001, ∗∗∗∗ *p* ≤ 0.0001.

Comparison of the colonic macrophage gene sets revealed clearly distinct transcriptional programs in the two genotypes. WD-fed RAG-2-deficient mice displayed a macrophage signature enriched for genes associated with epithelial barrier perturbation, mucosal remodeling and tissue repair. This included epithelial-associated genes such as *Epcam*, *Krt8*, *Muc2*, *Muc13*, *Cldn7*, *Pigr*, and *Retnlb*, together with extracellular matrix and wound-healing genes, including *Col1a1*, *Col1a2*, *Col3a1*, *Col4a1*, *Lamc1*, *Postn*, *Loxl2*, and *Serpinh1*. These findings are consistent with a macrophage population exposed to, or responding to, epithelial stress and tissue remodeling in the context of intestinal immune activation and metabolic disease. By contrast, macrophages from RAG-2/β7-integrin double-deficient mice showed increased expression of genes related to antigen uptake and processing, cellular migration, and immune surveillance, including *Zfp366*, *Ccr7*, *Wdfy4*, *Cd209a*, and *Irf7*. This transcriptional profile is compatible with a more immune regulatory and antigen-handling phenotype rather than with pronounced tissue injury and extracellular matrix remodeling. A similar distinction was observed in small intestinal macrophages. *Cd209a*, *Ccr7*, and *Tnfaip3* were higher expressed in RAG-2/β7-integrin double-deficient mice, suggesting enrichment of mononuclear phagocytes with migratory, antigen-presenting, and inflammation-regulatory properties. In contrast, *Reg3g* expression was increased in RAG-2-deficient mice, potentially reflecting enhanced antimicrobial and epithelial barrier-associated signaling.

Since a mechanistic link is thought to exist between WD-induced macrophage inflammation, Akt/mTOR signaling, and metabolic derangements such as glucose tolerance,^8^^,^^33^ we next used FACS analysis to investigate the influence of β7 integrin on the activation of the Akt signaling pathway in colonic (Figures 8D–8F) and small intestinal (Figures S9A–S9C) monocytes and macrophages. 4-week WD feeding caused a significant downregulation in mTOR phosphorylation of colonic macrophages, with no difference between genotypes (Figure 8D). In contrast, phosphorylation of AKT and S6 was significantly lower in colonic macrophages of WD treated RAG-2/β7-integrin double-deficient mice compared to colonic macrophages of RAG-2-deficient animals treated in the same way (Figures 8E and 8F). In addition, MALDI-MSI analysis showed significantly less pAkt in the colon of 24-week WD-treated RAG-2/β7-integrin double-deficient mice than in RAG-2-deficient mice (Figure S9D). In the small intestine, Ly6C^+^ CD64^+^ monocytes and macrophages showed increased phosphorylation of AKT and ribosomal protein S6 in response to WD feeding, whereas mTOR phosphorylation remained unchanged (Figures S9A–S9C). No significant genotype-dependent differences were detected in this compartment. These findings suggest that β7 integrin is involved in the regulation of downstream signaling pathways in colonic macrophages, in particular in the phosphorylation of ribosomal protein S6.

## Discussion

MetS is a systemic disease caused by the complex and not yet fully understood interactions of imbalanced pro-inflammatory immune cell populations with inflammatory signals derived from key metabolic organs including adipose tissue, liver, gut and the enteric microbiota.^2^^,^^3^ A prerequisite for the development of new therapies is a better understanding of these underlying mechanisms.

We set up experiments to better define the role of the adhesion molecule β7 integrin in WD-induced MetS. Previously, β7 integrin expressing T cells have been shown to promote diet-induced metabolic dysregulation.^20^^,^^21^^,^^22^ Here, we demonstrate that loss of β7 integrin or its interaction partner MAdCAM-1 protects RAG-2-deficient mice from WD-induced glucose intolerance and increases in body weight, EWAT weight, and serum cholesterol, suggesting that β7 integrin expressing innate immune cells contribute to manifestations of MetS.

The protective effect of the β7 integrin deficiency in this system is most likely mediated by a number of different mechanisms. On the one hand, lymphopenic mice with additional β7 integrin-deficiency were characterized by an increased RER, indicating a reduced utilization of fat as a food source. This is probably due to the observed altered intestinal lipid handling of such animals. Altered lipid handling may have various causes including changes in the fat-absorbing enterocytes of the small intestine. In this regard, a reduction in *Ces1D* expression, and a trend toward downregulated expression of other genes involved in lipid homeostasis such as *Pparγ* and *Srebp2*, which we observed on mRNA level in the intestines of anti-β7 integrin antibody-treated animals, may have contributed also to altered fat absorption in enterocytes of WD-treated RAG-2/β7 integrin double-deficient mice. The key hydrolase CES1D is responsible for catabolizing diet-derived cytoplasmic lipid droplets and its downregulation may disturb enterocyte lipid processing.^34^ An indirect influence of β7 integrin on the function of enterocytes has already been reported, β7 integrin having been shown to regulate the migration properties of enterocytes, probably via the interactions of αEβ7 integrin-expressing leukocytes with epithelial E-cadherin.^35^ Further investigation into the interaction between β7 integrin-expressing immune cells and enterocytes is warranted.

On the other hand, we could show that under the conditions of experimental induced obesity the colon, small intestine, and EWAT of RAG-2/β7 integrin double-deficient mice and mice treated with an anti-β7 integrin antibody had comparatively fewer monocytes and that β7 integrin-deficient monocytes have a reduced capacity to migrate into the colon of both RAG-2-deficient and CCR2-deficient animals. Furthermore, we found that in contrast to β7 integrin-deficient monocytes, WT monocytes caused a deterioration of the metabolic parameters of CCR2-deficient mice. Thus, we concluded that the migration of β7 integrin-expressing monocytes into colon and EWAT contributes to WD-induced MetS. This hypothesis is supported by data demonstrating that obesity and diabetes are associated with increased macrophage infiltration into the colon and that eliminating colonic macrophages improves glycemic control in diet-induced obesity.^6^^,^^7^^,^^8^ In addition, increased pro-inflammatory macrophages in adipose tissue were shown to exacerbate systemic metabolic disease in experimental obesity models, while interventions decreasing their numbers or shifting polarization improved glucose homeostasis and decreased adipose inflammation.^36^^,^^37^^,^^38^^,^^39^ Our data are also in line with previous reports on mice with a mutation in α4 integrin, which can exist as either α4β1 integrin or α4β7 integrin.^40^ These mice are protected against high-fat diet-induced insulin resistance, an effect attributed to reduced monocyte migration into adipose tissue. Our data suggest that this protective effect was caused by the absence of the α4β7 integrin heterodimer. While our findings implicate β7 integrin expressing monocytes in the pathogenesis of diet-induced MetS, studies incorporating subset-specific analysis of further β7 integrin-expressing innate immune cell populations that might contribute to the observed metabolic phenotype will be an important direction for future work.

The phenotype of lamina propria macrophages is strongly shaped by the local microenvironment, including epithelial damage, microbial products, dietary metabolites, and cytokine signals.^7^^,^^41^^,^^42^ Bulk RNA sequencing indicated that β7 integrin deficiency is associated with distinct intestinal macrophage transcriptional profiles during WD-induced MetS. In RAG-2-deficient mice, which developed more severe metabolic disease, colonic macrophages exhibited increased expression of genes related to epithelial barrier function, extracellular matrix organization, and tissue repair. This profile is consistent with macrophage responses to epithelial stress and tissue remodeling, reflecting the established contribution of intestinal macrophages to barrier maintenance, inflammatory regulation, and mucosal repair.^43^ By contrast, macrophages from RAG-2/β7 integrin double-deficient mice showed higher expression of genes associated with antigen uptake and presentation, cellular migration, and negative regulation of inflammation, including *Cd209a*, *Ccr7*, *Wdfy4*, *Zfp366*, *Irf7*, and *Tnfaip3*. These findings are compatible with a mononuclear phagocyte population that remains immunologically vigilant but is better equipped to regulate inflammatory activation, consistent with the established roles of CCR7 in intestinal immune-cell migration and TNFAIP3/A20 in restraining inflammatory signaling.^44^^,^^45^ Regional differences, such as lower microbial density could be responsible for the more restricted pattern of differences seen in small intestinal macrophages,^41^ involving a higher *Cd209a*, *Ccr7*, and *Tnfaip3* expression in RAG-2/β7 integrin double-deficient mice, and higher *Reg3g* expression in RAG-2-deficient mice. Reg3g is linked to microbial exposure and epithelial stress and its increased expression might reflect a compensatory response to barrier disruption or increased microbial translocation in the metabolically affected mice.^46^ However, because several differentially expressed genes are also characteristic of epithelial, stromal, or dendritic-cell-like populations, the observed signatures may also reflect alterations in the macrophage-associated niche or shifts in mononuclear phagocyte composition rather than macrophage-intrinsic reprogramming alone.

Furthermore, we observed a comparatively reduced AKT and S6 phosphorylation in β7 integrin-deficient colonic macrophages. A number of recent publications have suggested that the Akt pathway in colonic and adipose tissue macrophages is crucial for integrating nutrient and inflammatory signals in obesity, promoting a pro-inflammatory state that drives metabolic dysregulation.^47^ In this context, using a diet-induced obesity model, Rohm et al. have shown an improvement in insulin secretion capacity upon colon-specific mTOR inhibition similar to that achieved by colon-specific macrophage depletion.^8^ The MetS promoting role of β7 integrin regarding macrophages therefore appears not only to be limited to its role in driving colonic immigration but also in influencing the endogenous functions of pro-inflammatory macrophages. More detailed studies including cell-specific deletion of β7 integrin in the context of disease development and dissection of intracellular cell signaling after ligand binding and cell-cell contacts are needed to further elucidate the roles of β7 integrin-expressing macrophages in metabolic dysregulation.

The intestinal microbiome and its metabolites contribute to the maintenance of the intestinal barrier and immune homeostasis. WD is known to alter the gut microbiota structure and may thereby compromise gut integrity, activate innate immunity and trigger systemic inflammation.^48^^,^^49^ In this context it was reported that certain microbial metabolites can upregulate the αEβ7 integrin binding partner E-cadherin in the intestinal epithelium and that antibiotic-induced dysbiosis suppresses MAdCAM-1 expression via changes in bile acid metabolism.^50^^,^^51^ In our study, antibody-mediated inhibition of β7 integrin during the 4-week WD feeding period caused only minor alterations in fecal microbiota profiles. The WD-induced reduction in relative abundances of *Muribaculaceae*^52^ and *Prevotellaceae*,^53^ which was observed independent of the antibody treatment, corroborates former studies.^54^^,^^55^ The proportions of *Erysipelotrichaceae* and *Peptostreptococcaceae* were higher in feces of WD-fed RAG-2-deficient mice treated with the isotype control antibody, than in mice that had received the anti-β7 integrin antibody FIB 504. An increased representation of *Erysipelotrichiaceae* and *Peptostreptococcaceae* in feces of WD fed mice has also been reported previously.^56^^,^^57^ *Peptostreptococcaceae* contribute to cholesterol conversion and might contribute to WD-induced hypercholesterolemia and supplementation of mice with *Romboutsia ilealis (Peptostreptococcaceae)* has been shown to lead to impaired glucose tolerance.^58^^,^^59^ In contrast, β7 integrin inhibition was associated with increased *Lachnospiraceae*, key short-chain fatty acid producers previously linked to metabolic benefits in FMT studies.^60^ Overall, antibody-mediated blocking of β7 integrin during WD feeding exerts beneficial metabolic effects that correlate with minor shifts in fecal microbiota profiles.

In summary, we have shown that the β7 integrin-mediated manifestation of MetS is also supported by cells of the innate immune system, in addition to lymphocytes, and that this regulation might occur at various levels. This includes β7 integrin-directed migration of monocytes into metabolically active tissues such as fat and intestine, changes in macrophage signaling, and in intestinal lipid handling, and points to the potential of a cell-type specific targeting of β7 integrin signaling for treatment of MetS.

### Limitations of the study

Our study identifies β7 integrin-expressing monocytes as key contributors to the pathogenesis of diet-induced MetS, while also highlighting several important avenues for future investigation. First, subset-specific analyses of other β7 integrin-expressing innate immune cell population will be valuable to determine their respective contributions to the observed metabolic phenotype. Further exploration of the crosstalk between β7 integrin-expressing immune cells and enterocytes will also provide important mechanistic insights. Second, although our data are consistent with the hypothesis that altered intestinal lipid handling underlies protection against WD-induced MetS, the current findings remain correlative. Establishing causality will require additional measurements, such as fecal caloric loss or direct intestinal triglyceride absorption assays, which were beyond the scope of this study. Finally, metabolic cage analyses assessing energy expenditure and substrate utilization will be instrumental in defining the extent to which β7 integrin-expressing monocytes shape the overall metabolic phenotype.

## Resource availability

### Lead contact

Requests for further information and resources should be directed to and will be fulfilled by the lead contact, Angela Schippers (anschippers@ukaachen.de).

### Materials availability

This study did not generate new unique reagents.

### Data and code availability

• Data files from the bulk RNA sequencing analysis have been uploaded to the European Nucleotide Archive (ENA) and are publicly available under the accession number PRJEB111193.
• The microbiota sequencing data presented in this study have been uploaded to the European Nucleotide Archive (ENA) and are publicly available under the accession number PRJEB111158.
• This study does not report original code.
• Any additional information required to reanalyze the data reported in this study is available from the lead contact upon request.

## Acknowledgments

This research was funded by the 10.13039/501100001659DFG, Gemany (German Research Foundation)–Project-ID 403224013–SFB 1382 to T.C. (subproject Q02), N.W. (subproject B02), J.J.(subproject A04), and A.S. (subproject B02) and SFB/TRR 219; Project-ID 322900939 to J.J. (subproject S-03 and C-04, INST 948/4S-1 FUGG & INST 222/1510-1-FUGG). This work was also supported by COST Action PerMedik, CA21165, supported by COST (European Cooperation in Science and Technology) COST to J.J. and by a grant from the Interdisciplinary Centre for Clinical Research within the faculty of Medicine at the RWTH Aachen University to J.J. (subproject PTD 1-1). We also received support by the “Immunohistochemistry facility,” a core facility of the Interdisciplinary Center for Clinical Research (10.13039/501100009379IZKF, Germany) Aachen within the Faculty of Medicine at RWTH Aachen University.

## Author contributions

Conceptualization, S.E., A.S., and N.W.; formal analysis, S.E., A.Se., N.T., T.C., K.S., S.H., J.H., T.-H.N., and A.S.; funding acquisition, T.C., J.J., N.W., and A.S.; investigation, S.E., A.Se., N.T., T.C., K.S., S.H., J.H., T.-H.N., and A.S.; project administration, S.E. and A.S.; resources, T.C., J.J., and N.W.; supervision, A.Se. and N.W.; validation, S.E., A.Se., N.T., T.C., K.S., S.H., T.-H.N., J.J., J.H., N.W., and A.S.; writing – original draft, S.E. and A.S.; writing – review and editing, S.E., A.Se., N.T., T.C., K.S., S.H., J.H., T.-H.N., J.J., N.W., and A.S..

## Declaration of interests

The authors declare no competing interests.

## STAR★Methods

### Key resources table

**Table:** 

REAGENT or RESOURCE	SOURCE	IDENTIFIER
**Antibodies**		

FITC rat monoclonal anti-mouse CD103 antibody, clone M290	BD Biosciences	Cat# 557494; RRID:AB_396731
Biotinylated rat monoclonal anti-mouse CD115 antibody, clone AFS98	Invitrogen	Cat# 13-1152-85; RRID:AB_466565
BV510 rat monoclonal anti-mouse CD11b antibody, clone M1/70	BD Biosciences	Cat# 562950; RRID:AB_2737913
APC-Cy7 rat monoclonal anti-mouse CD45 antibody, clone 30-F11	eBioscience	Cat# 557659; RRID:AB_396774
eFluor450 mouse monoclonal anti-mouse CD45.1 antibody, Clone A20	eBioscience	Cat# 48-0453-82; RRID:AB_1272189
FITC mouse monoclonal anti-mouse CD45.2 antibody, Clone 104	eBioscience	Cat# 11-0454-82; RRID:AB_465061
BV421 mouse monoclonal anti-mouse CD64 antibody, Clone X54-5/7.1	BioLegend	Cat# 139309; RRID:AB_2562694
FITC mouse monoclonal anti-mouse CX3CR1 antibody, clone SA01F11	BioLegend	Cat# 149019; RRID:AB_2565702
PE mouse monoclonal anti-mouse CX3CR1 antibody, clone SA01F11	Biolegend	Cat# 149005; RRID:AB_2564314
PB rat monoclonal anti-mouse F4/80 antibody, Clone CI:A3-1	Bio Rad	Cat# MCA497PB; RRID:AB_567114
PE rat monoclonal anti-mouse integrin β7 chain antibody, Clone M293	BD Biosciences	Cat# 557498; RRID:AB_396735
PerCP-Cy5.5 rat monoclonal anti-mouse Ly-6C antibody, clone AL-21	BD Biosciences	Cat# 560525; RRID:AB_1727558
FITC rat monoclonal anti-mouse Ly-6G antibody, clone 1A8	BD Biosciences	Cat# 561105; RRID:AB_10562567
PE-Cy7 mouse monoclonal anti-mouse Phospho-mTOR (Ser2448) antibody, clone MRRBY	eBioscience	Cat# 25-9718-42; RRID:AB_2573550
PE mouse monoclonal anti-mouse Akt (pS473) antibody, clone M89-61	BD Biosciences	Cat# 560378; RRID:AB_1645328
Alexa Fluor 488 mouse monoclonal anti-mouse S6 (pS235/pS236) antibody, clone N7-548	BD Biosciences	Cat# 560434; RRID:AB_2869347
Rabbit polyclonal anti-mouse CD68 antibody	Abcam	Cat# 125212; RRID:AB_10975465
Biotinylated goat polyclonal anti-rabbit IgG antibody (H&L)	Vector Laboratories	Cat# BA-1000; RRID:AB_2313606
PerCP/Cy5.5 rat monoclonal anti-human/mouse integrin β7 chain antibody, Clone FIB27	Biolegend	Cat# 121008; RRID: AB_2129312
BV421 rat monoclonal anti-mouse Ly-6C, Clone HK1.4	Biolegend	Cat# 128032; RRID: AB_2562178
APC rat monoclonal anti-mouse I-A/I-E, Clone M5/114.15.2	Biolegend	Cat# 107613; RRID: AB_313328
FITC armenian hamster monoclonal anti-mouse CD11c, Clone N418	eBioscience	Cat# 11-0114-82; RRID: AB_464940

**Chemicals, peptides, and recombinant proteins**		

Poloxamer P407	Sigma-Aldrich	Cat# 16758
α-cyano-4-hydroxycinnamic acid	Bruker Daltonics	Cat# 8201344

**Critical commercial assays**		

Zombie red fixable viability kit	BioLegend	Cat# 423109
LegendPlex mouse inflammation panel (13-plex)	BioLegend	Cat# 740446
Ultra-sensitive mouse insulin ELISA	Crystal Chem	Cat# 90080
LabAssay Triglyceride	FUJIFILM Wako	Cat#291-94501
LabAssay Cholesterol	FUJIFILM Wako	Cat#293-93601
Free Fatty Acid Assay kit	Abcam	Cat# ab65341
Vectastain ABC elite kit	Vector Laboratories	Cat# PK-6100
DAB substrate kit	Vector Laboratories	Cat# SK-4100
RNeasy Protect Mini Kit	Qiagen	Cat# 74126
RNase-free DNase Set	Qiagen	Cat# 79256
Transcriptor FS cDNA synthesis-kit	Roche	Cat# 48970300001
iTaq^TM^ Universal SYBR®Green Supermix	Bio Rad	Cat# 172-5125
QuantSeq 3′ mRNA-Seq V2 Library Prep Kit with UDI	Lexogen	Cat# SKU 191.24
UMI Second Strand Synthesis Module for QuantSeq FWD	Lexogen	Cat# SKU 081.96
ERCC RNA Spike-In Mix	Thermo Fisher	Cat# 4456740
NextSeq 500/550 High Output Kit v2.5 (75 Cycles)	Illumina	Cat# 20024906

**Deposited data**		

Microbiota sequencing data	This paper	European Nucleotide Archive (ENA); PRJEB111158
Fastq files from bulk RNA sequencing analysis	This paper	European Nucleotide Archive (ENA); PRJEB111193

**Experimental models: Organisms/strains**		

C57BL/6J mice	In house breeding from mice that were originally derived from Janvier.	N/A
CCR-2-deficient (CCR2^−/−^) mice	Boring, L. et al. *J Clin Invest***100**, 2552–2561 (1997).^32^	N/A
RAG-2-deficient (RAG-2^−/−^) mice	Shinkai, Y. et al. *Cell***68**, 855–867 (1992).^26^	N/A
β7 integrin/RAG-2 double-deficient (RAG-2^−/−^/β7^−/−^) mice	Originated from crossing of RAG-2^−/−^ and β7^−/−^mice^14^	N/A
MAdCAM-1/RAG-2 double-deficient (RAG-2^−/−^/MADCAM-1^−/−^) mice	Originated from crossing of RAG-2^−/−^ and MAdCAM-1^-/-^mice^15^	N/A

**Oligonucleotides**		

***Gapdh forward:*** acctgccaagtatgatgacatca ***Gapdh*****reverse:** ggtcctcagtgtagcccaagat	This paper	N/A
***Ccl2* forward:** gtgttggctcagccagatgc***Ccl2* reverse:** gacacctgctgctggtgatcc	This paper	N/A
***Adgre1*forward:** ctctgtggtcccaccttcat ***Adgre1* reverse:** gatggccaaggatctgaaaa	This paper	N/A
***Cd68* forward:** cttcccacaggcagcacag ***Cd68* reverse:** aatgatgagaggcagcaagagg	This paper	N/A
***Ces1d*forward:** atatggctttctcttgctgcg ***Ces1d*reverse:** cccaggactttgcctttaacagt	This paper	N/A
***Srebp1a*forward:** ggccgagatgtgcgaact ***Srebp1a* reverse:** ttgttgatgagctggagcatgt	This paper	N/A
***Srebp2*forward:** gcgttctggagaccatgaa ***Srebp2* reverse:** acaaagttgctctgaaaacaaatca	This paper	N/A
***Pparg*forward:** tgtcggtttcagaagtgccttg ***Pparg*reverse:** ttcagctggtcgatatcactggag	This paper	N/A

**Software and algorithms**		

TSE PhenoMaster (version 5.3)	TSE Systems	https://tse-phenomaster.software.informer.com/
Data analysis software suite for LEGENDplex	BioLegend	https://biolegend.com
ZEISS ZEN lite	ZEISS	https://www.zeiss.com/microscopy/de/produkte/software/zeiss-zen-lite.html
SCiLS Lab software (SCiLS Lab-2025b)	BRUKER	https://www.bruker.com/en/products-and-solutions/mass-spectrometry/ms-software/scils-lab.html
BioTools 3.2	Bruker Daltonics	https://bruker-daltonics-biotools.software.informer.com/3.2/
IMNGS	Lagkouvardos, I. et al.^61^	https://www.imngs.org/
Rhea	Lagkouvardos, I. et al.^62^	https://github.com/Lagkouvardos/Rhea
FlowJo (version10.2)	BD Biosciences	https://flowjo.com
GraphPad Prism (version 10)	GraphPad	https://www.graphpad.com
Biorender	BioRender	https://biorender.com
CFX Opus 96 Real-Time PCR System	Bio-Rad	https://www.bio-rad.com/de-de/sku/12011319-cfx-opus-96-real-time-pcr-system?ID=12011319
bcl2fastq Conversion software	Illumina	https://support.illumina.com/sequencing/sequencing_software/bcl2fastq-conversion-software.html
CalR	Mina, A. et al.^63^	https://calrapp.org/#
R software	R core team (2025)	https://www.r-project.org/
RStudio	Posit team (2025)	https://posit.co/download/rstudio-desktop/
FastQC	Simon Andrews	https://www.bioinformatics.babraham.ac.uk/projects/fastqc/
MultiQC	Ewels, P. et al.^64^	https://github.com/MultiQC/MultiQC
Salmon	Patro, R. et al.^65^	https://salmon.readthedocs.io/en/latest/index.html
edgeR	Robinson, M. et al.^66^	https://bioconductor.org/packages/release/bioc/html/edgeR.html

**Other**		

7-AAD Viability staining solution	BioLegend	Cat# 420403
APC Calibrite counting beads	BD Biosciences	Cat# 340487
eFluor 450 Cell proliferation dye	eBioscience	Cat# 65-0842-85
eFluor 670 Cell proliferation dye	eBioscience	Cat# 65-0840-85
Chow diet	ssniff, Soest, Germany, rat/mouse–maintenance	Cat# V1535-000
Western diet	Brogaarden, Lynge, Denmark	Cat# D16022301
50% Glucose	B. Braun	Cat# 04164543
100 U/mL Insulin (NovoRapid)	Novo Nordisk, Bagsværd	PZN06607111
RNAprotect tissue reagent	Qiagen	Cat# 76106
gentleMACS M Tubes	Miltenyi	Cat#130-096-335
Pharm lyse lysing buffer	BD Pharmingen	Cat# 555899
Anti-biotin microbeads	Miltenyi	Cat# 130-097-046
MACS LS Separation Column	Miltenyi	Cat#130-042-401
Collagenase V	Sigma-Aldrich	Cat# C9263
Collagenase D	Roche	Cat# 11088882001
Dispase	GIBCO	Cat# 17105-041
DNase I G-II	Roche	Cat# 10104159001
HBSS	Invitrogen	Cat#14170-088
FCS	ThermoFisher Scientific	Cat# 10500-064
RPMI 1640 medium	ThermoFisher Scientific	Cat# 21875-034
Ficoll	Pan Biotech	Cat# P04-601000

### Experimental model and study participant details

Animals were bred at RWTH Aachen University and housed in individually ventilated cages under specific pathogen-free conditions with 12 h light/dark cycles and water and food available *ad libitum* and received autoclaved wood chip bedding (Lignocel), tunnel, and nestlet enrichment. Experiments were performed with age-matched 8 to 15-week-old C57BL/6J mice, RAG-2-deficient (RAG-2^−/−^) mice,^26^ β7 integrin/RAG-2-double-deficient (RAG-2^−/−^/β7^−/−^) mice,^14^ MAdCAM-1^−/−^/RAG-2^−/−^ double-deficient mice,^15^ and CCR-2-deficient (CCR2^−/−^)^32^ mice of similar weight on C57BL/6J background from the same barrier and room. Only male mice were used in this study as female mice are more resistant to metabolic disorders and to control for the confounding effects of sexual dimorphism. Therefore, sex-dependent effects were not evaluated, which is a recognized limitation. All efforts were made to minimize the number of animals used and their suffering. The experimental protocols were approved by the competent state licensing authorities for animal experimentation, State Office for Consumer Protection and Nutrition of North Rhine-Westphalia ((LAVE, Recklinghausen, Germany), project licenses 81-02.04.2023. A215 and 81-02.04-2021.A402). All experiments were performed in accordance with the European Directive 2010/63/EU, the German Animal Welfare Act, and the relevant guidelines issued by LAVE. Procedures were reviewed and approved by the institutional animal welfare body (animal welfare commission, animal welfare officer) of the RWTH Aachen University, and authors complied with the ARRIVE guidelines.

### Method details

#### Dietary treatments

Mice were fed chow (9 kcal % fat, 24 kcal % protein, 67 kcal % carbohydrates) (ssniff, Soest, Germany, rat/mouse–maintenance) or WD (40 kcal % fat, mostly Non Trans Fat Primex, 20 kcal % fructose, 2% cholesterol) (Brogaarden, Lynge, Denmark; cat. no. D16022301). Experimental mice were kept in single cages, which were changed weekly, and provided with fresh food and water. Body weight and food intake were assessed weekly over the duration of 4 or 24 weeks of WD treatment. Food intake was calculated as the difference between grams of food added and grams of food remaining in the cage. At the end of the experiment, the animals were sacrificed by CO_2_ inhalation and subsequent cervical dislocation. All the experiments were repeated in at least two independent experimental setups.

#### Intraperitoneal glucose and insulin tolerance test (GTT and ITT)

Mice were fasted for 6 h and blood glucose was measured using an Accu-Check® Aviva meter (Roche, Basel, Switzerland), via one drop of blood taken from the animal’s tail, every 15 min for 2 h following intraperitoneal administration of 2 g/kg glucose (B. Braun#04164543) or 0.5 U/kg insulin (NovoRapid, Novo Nordisk, Bagsværd).

#### Oral fat tolerance test

Mice were fasted for 6 h and injected intraperitoneally with 1 g/kg poloxamer (P407 Sigma 16758) and gavaged with olive oil (10μl/g bodyweight). Mice were sacrificed directly, 2 hours or 6 hours after gavage.

#### Metabolic cages

Oxygen consumption (VO2) and carbon dioxide expiration (VCO2) were measured using the PhenoMaster cage system (TSE Systems, Germany) following the manufacturer’s instructions. Mice were acclimatized for 6 days prior to 3 days of actual measurements.

#### Serum measurements

Glucose, triglyceride, and cholesterol levels in serum were measured by the Central Laboratory Facility of the University Hospital, RWTH Aachen.

Serum cytokine concentrations were measured by a bead-based immunoassay technique using the LegendPlex Mouse Inflammation Panel (13-plex) (Biolegend, San Diego, cat. no 740446) following the manufacturer’s instructions. Samples were acquired on a Canto-II flow cytometer (BD Biosciences) and data were analyzed using the LegendPlex data analysis software.

#### Fecal lipid extraction and quantification

Fecal lipids were extracted using the method of Folch et al.^67^ and quantified using colorimetric assays. Briefly, fecal samples were collected at baseline (chow diet) and after 4 weeks of Western diet (WD) treatment from individually housed mice. Fecal aliquots were dried for 1 hour at 70°C and incubated in 1 mL chloroform:methanol (2:1, v/v) for 30 minutes at room temperature with constant agitation. Samples were subsequently mechanically homogenized and centrifuged to remove insoluble material. Water (500 μL) was added to the supernatant, followed by vigorous shaking and centrifugation at 2,000 × g for 10 minutes to induce phase separation. The lower chloroform phase was collected, transferred to a fresh tube, and evaporated to dryness. The dried lipid extracts were resuspended in 500 μL chloroform containing 1% Triton X-100, evaporated to dryness, and subsequently resuspended in 500 μL water, resulting in a final solvent composition of 1% Triton X-100 in water.

Total cholesterol (Wako, Neuss, Germany), triglycerides (Wako, Neuss, Germany), and free fatty acids (Abcam, United Kingdom) were quantified in the fecal lipid extracts using enzymatic colorimetric assay kits according to the manufacturers’ instructions. Corresponding assay standards were prepared in 1% Triton X-100 in water to match the solvent composition of the samples.

#### Histological stainings

All stainings were performed on 4 μm paraffin sections. Images were acquired using an Axioplan2 microscope (Carl Zeiss Microscopy, Oberkochen, Germany) and Zen lite 3.2 software (Carl Zeiss Microscopy, Oberkochen, Germany). For immunohistochemical staining of CD68, formalin-fixed tissue sections were first permeabilized. After heat-induced epitope retrieval in TE buffer pH 9.0, the sections were blocked with 2% goat serum and incubated with a rabbit-anti-CD68 antibody overnight at 4 °C. The sections were then treated with biotinylated goat-anti-rabbit IgG for 30 min at room temperature. Antibody binding was visualized with the ABC kit and diaminobenzidine reagent as a chromogen (both from Vector, Burlingame, USA). Nuclei were stained with hematoxylin. Antibodies are listed in the key resources table.

#### RNA isolation and cDNA synthesis

Tissues were stored in RNA-Protect® at -20 °C until processing. Total RNA was extracted from samples using the RNeasy kit including a DNA digestion step with the RNase Free DNAse set (both from Qiagen GmbH, Hilden, Germany) according to the manufacturer’s description. The tissue was homogenized in gentleMACS M Tubes using the RNA_02.01 setting on the gentle MACS dissociator (Miltenyi Biotec, Bergisch Gladbach, Germany). RNA concentration was photometrically measured, and integrity was checked on a Nanodrop ND-100 (Thermo Fisher Scientific, USA). cDNA was synthesized from 1 μg RNA using the Transcriptor FS cDNA S-Kit (Roche Diagnostic GmbH, Mannheim, Germany) following the manufacturer’s instructions.

#### Gene expression analysis by real-time PCR

Real-time polymerase chain reactions (RT-PCR) were performed in duplicate on a Bio-Rad CFX Opus 96 RT-PCR System, with Bio-Rad CFX Maestro software version 2.3 using the iTaq^TM^ Universal SYBR®Green Supermix (all from Bio-Rad Laboratories GmbH, Feldkirchen, Germany). Glyceraldehyde 3-phosphate dehydrogenase (GAPDH) was used as endogenous control for normalization). Primer sequences are listed in the key resources table.

#### Flow cytometry

All flow cytometric measurements were performed on a Canto-II cytometer (BD Biosciences). Single cell suspensions were stained directly using combinations of the monoclonal antibodies listed in the key resources table. Representative FACS dot plots illustrating the gating strategy are shown in Figures S10–S13. Data were analyzed by FlowJo 10.2 software (Tree Star, Ashland, OR, USA).

#### Isolation of bone marrow monocytes for adoptive cell transfer

Single cell suspensions from bone marrow of WT and β7 integrin-deficient donors were obtained by flushing ice cold Phosphate Buffered Saline (PBS)/0.5 % Bovine Serum Albumin (BSA) through the open ends of excised and cleaned femurs and filtering through a 40-μm cell strainer. For the comparative migration analysis of adoptively transferred bone marrow cells, cell pellets were lysed with Erylysis buffer (BD Pharmingen, San Jose, CA), washed with PBS and labeled with cell proliferation dye (efluor 670 and efluor 450) according to the manufacturer’s instructions. Equal numbers of differently labeled WT and β7 integrin-deficient cells were intravenously transferred into 4-week WD-fed RAG-2^−/−^ and CCR2^−/−^ mice. Each mouse received 1-3 x 10^7^ total cells in 100 μl sterile PBS via tail vein injection. 16 hours after the transfer, the mice were sacrificed and the migratory capacity of the labeled cells into different organs was compared by flow cytometry.

To compare the activity of monocytes in promoting MetS, CD115^+^ monocytes were isolated from bone marrow by magnetic cell separation (MACS; Miltenyi Biotech) according to the manufacturer’s instructions. Briefly, unlysed bone marrow single-cell suspensions were pooled and CD115^+^ monocytes were purified by MACS using a biotinylated anti-CD115 antibody, followed by anti-biotin microbeads and separation via MACS columns according to the manufacturer’s instructions. Purity of the isolated cell fractions was controlled by flow cytometry. The cells were resuspended in an appropriate volume of sterile PBS allowing for around 5.5 x 10^6^ per tail vein injection per CCR2^-/-^ recipient, once at the beginning of the diet treatment and a second time after 2 weeks.

#### Matrix-assisted laser desorption/ionization (MALDI) imaging data acquisition and data analysis

Matrix-assisted laser desorption/ionization (MALDI) techniques were used to analyze the intra-intestinal attendance of distinct proteins in chow and WD-fed mouse small intestine and colon. The sections of the paraffin-embedded tissue specimens were deparaffinized and coated with a trypsin solution (25 ng/μl in 20 mM ammonium bicarbonate), incubated for 2 h at 50°C and coated with α-cyano-4-hydroxycinnamic acid (Bruker Daltonic, Germany) as matrix (10 mg/ml dissolved in 70 % ACN/ 0.2 % TFA). The coating process was performed by using a sprayer for MALDI imaging (HTX TM-Sprayer: TMSP-M3, HTX Technologies, USA). MALDI MSI data accumulation was performed using a TOF-TOF-mass spectrometer (Rapiflex; Bruker-Daltonic, Bremen, Germany) equipped with a SmartbeamTM 3D laser with a 10 kHz repetition rate and controlled by the Flex-Control 4.0 (Bruker-Daltonic, Germany). Mass spectra were acquired with 800 laser shots for each raster point and a digitizer rate of 2.5 GS/s in reflector positive mode. Analyses were taken in mass range 0-3000 Da by using a raster width of 20 μm and spot diameter of 20 μm for colon samples and a raster width of 80μm and spot diameter of 80 μm for the small intestine.

Data analysis was performed using SCiLS Lab software (SCiLS Lab-2025b; SCiLS GmbH; Bremen, Germany). MALDI MSI raw data sets were converted to the SCiLS SL file format. The baseline was calculated by iterative top hat, and normalization was performed based on the total ion count (TIC) method. Representative mass signals (m/z) of pre-defined proteins were chosen for analyses of the distribution of the protein in the corresponding samples. The identification of peptides was performed through the MS/MS spectra using the lift option of the Rapiflex mass spectrometer (Bruker-Daltonic, Bremen, Germany). The mass spectra were calibrated and annotated using BioTools 3.2 (Bruker-Daltonic, Bremen, Germany) in combination with SwissProt (University of Geneva, Switzerland) and the MASCOT 2.2 database (Matrix Science, London, UK) comparing experimental mass-spectrometric data with calculated peptide masses for each entry into the sequence database.

#### DNA isolation from feces, 16S rRNA gene amplicon sequencing, and data analysis

DNA isolation from stool samples, library preparation, and sequencing were performed by the Functional Microbiome Research Group at the Faculty of Medicine, RWTH Aachen University, as previously described.^68^ Data analysis and statistics were performed using IMNGS^61^ and Rhea.^62^

#### Bulk RNA sequencing

Single cell suspensions from the colonic and small intestinal lamina propria were obtained by enzymatic digestion using Collagenase V (0.85 mg/mL; Sigma), Collagenase D (1.25 mg/mL; Roche), Dispase (1 mg/mL; GIBCO) and DNase (30 μg/mL; Roche) for a maximum of 45 min or until tissue was dissolved at 37°C. Cells were stained for CD45, SiglecF, Ly6G, CD11b, CD64 and 7AAD was added prior to acquisition (antibodies listed in the key resources table) and live CD45^+^ SiglecF^-^Ly6G^-^CD11b^+^CD64^+^ macrophages were sorted using an Aria Fusion cytometer (BD Biosciences). Sorted cells were stored in RNA-Later (Qiagen) at -80°C until analysis.

3′mRNA sequencing libraries were prepared using the Lexogen QuantSeq 3′mRNA-Seq FWD Library Prep Kit v2 with Unique Dual Indices (UDIs) and the UMI Second Strand Synthesis (USS) module, following the manufacturer’s instructions. ERCC RNA Spike-In Mix was added at a 1:100 dilution. Single-end sequencing (75 cycles) was performed on an Illumina NextSeq platform using the High Output v2.5 kit with a 20 % PhiX spike-in control.

Raw single-end FASTQ files were first assessed with ‘FastQC’ and summarized with ‘MultiQC’.^69^ Based on adapter content and end-quality metrics, reads were conservatively trimmed with ‘fastp’, followed by repeat FastQC/MultiQC^69^ assessment. Trimmed reads were quantified against the mouse Ensembl GRCm39 release 110 transcriptome using ‘Salmon’^65^ with a decoy-aware transcriptome index, to mitigate potential spurious mapping of reads. Transcript abundances were summarized to gene level with a transcript-to-gene map derived from the matching Ensembl annotation. ERCC features were removed, and Ensembl identifiers were annotated with gene names. Differential expression analysis was performed in R using ‘edgeR’,^66^ with lowly expressed genes filtered by ‘filterbyExpr’, library sizes normalized by TMM, and quasi-likelihood negative binomial generalized linear models fitted using an 8-group design. All unique pairwise comparisons were tested with ‘glmQLFTest’, and genes were considered differentially expressed at false discovery rate (FDR) < 0.05.

### Quantification and statistical analysis

#### Statistical analysis

For all experiments, n represents the number of mice per group. Statistical tests and *n* values are noted in the corresponding figure legends. Unless otherwise indicated, statistical analysis was performed with GraphPad Prism software (version 10 GraphPad, La Jolla, CA, USA). Flow cytometry data were analyzed using FlowJo software. Analysis of microbiota changes (calculation of alpha-diversity, beta-diversity and rarefaction curves) was performed in R using Rhea^62^ and statistical significance was calculated by two-way ANOVA and PERMANOVA for nMDS plots. For RNA-seq analysis, samples were evaluated using FastQC and MultiQC^64^ metrics, and one group was excluded due to failing quality thresholds. Differential expression analysis was performed on remaining samples and genes with a false discovery rate (FDR) < 0.05 were considered significantly differentially expressed. Data are presented as mean ± Standard Deviation (SD) or mean ± Standard Error (SEM). Significance values were calculated using the Student’s t-test when comparing two groups or one or two-way analysis of variance (ANOVA) and Tukey post-test. Values of *p* < 0.05 were considered significant (∗ *p* < 0.05, ∗∗ *p* < 0.01, ∗∗∗ *p* < 0.001, and ∗∗∗∗ *p* ≤ 0.0001).
