# Supplementary material for: β7-Integrin worsens diet-induced metabolic syndrome in mice by altering intestinal lipid handling and colonic inflammatory monocyte infiltration
Source: iScience. 2026 Oct 27;29(11):118042. doi: 10.1016/j.isci.2026.118042 (PMC13733324; doi:10.1016/j.isci.2026.118042)
Supplement: Supplementary file 1 [file mmc1.pdf]

iScience, Volume *29*

Supplemental information

β7-Integrin worsens diet-induced metabolic syndrome in mice by altering intestinal lipid handling and colonic inflammatory monocyte infiltration

Sreepradha Eswaran, Agnes Seeger, Sabine Hamm, Kerstin Scheuer, Nicole Treichel, Juliane Hermann, Trong-Hieu Nguyen, Thomas Clavel, Joachim Jankowski, Norbert Wagner, and Angela Schippers

Supplementary Figures

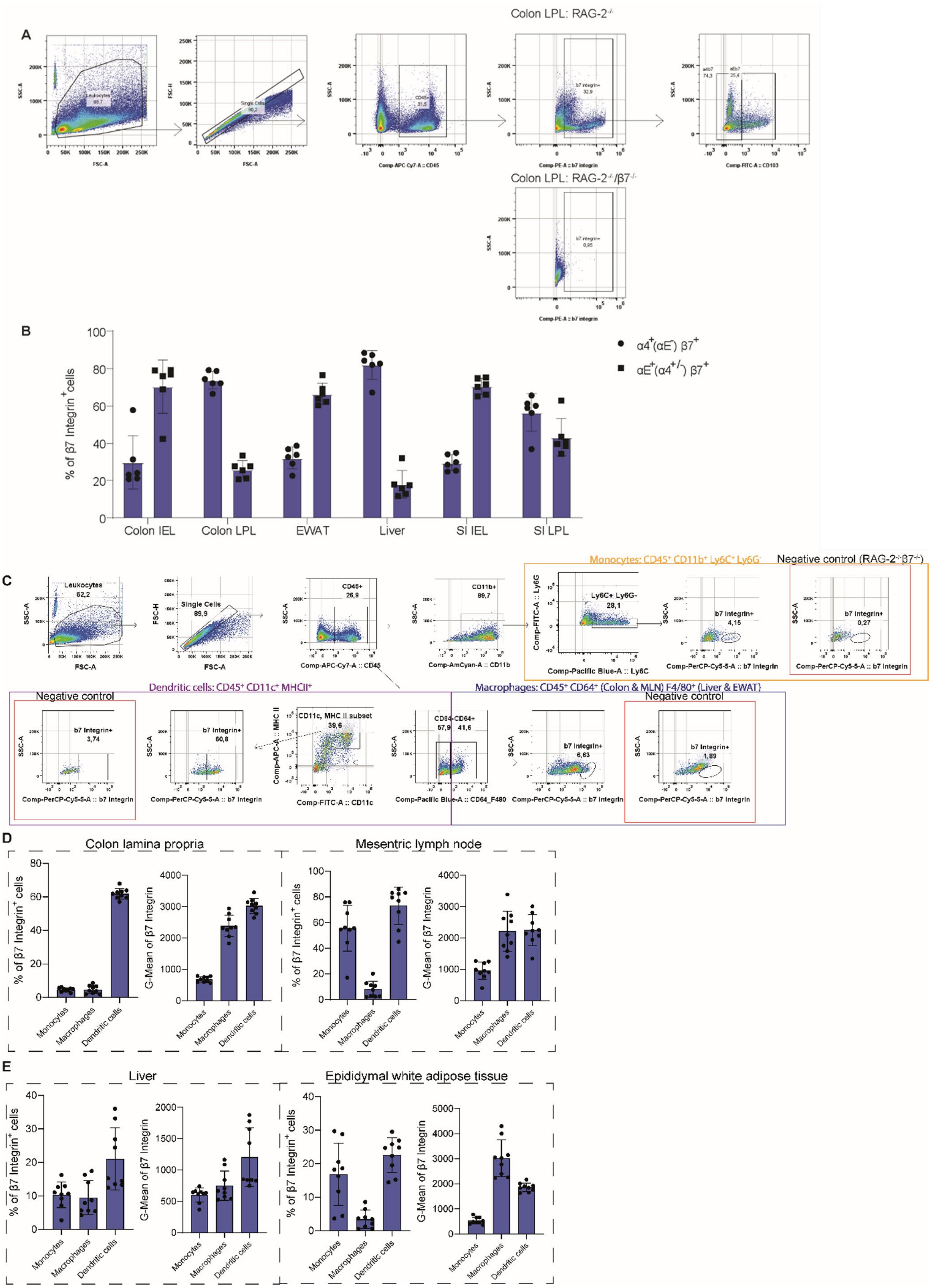

Figure S1. Flow cytometric analysis of β7 integrin expression. (A) Representative gating strategy for αE (CD103) and β7 integrin chains on cells from the colon lamina propria layer (LPL). As β7 integrin can exist only as the α4β7 or αEβ7 heterodimer, αE⁻β7⁺ stained cells are regarded as only α4β7 integrin positive. (B) β7 integrin positive cells (including αE⁺β7⁺ and α4⁺β7⁺ cells, shown as squares) and α4β7

integrin positive cells (including αE⁻β7⁺ cells, shown as circles) as a percentage of CD45⁺ cells of colon, small intestine (SI) intraepithelial layer (IEL) and lamina propria layer (LPL), liver, and epididymal white adipose tissue (EWAT) from chow diet (CD) fed RAG-2-deficient mice (n = 6). (**C**) Representative multiparameter flow cytometry gating strategies for β7 integrin expression on monocytes, macrophages and dendritic cells from the colonic lamina propria. All samples were first gated as leukocytes on FSC-A against SSC-A followed by exclusion of doublet cells by gating on FSC-A against FSC-H. Monocytes were defined as $CD45^+CD11b^+Ly6C^+Ly6G^-$, macrophages as $CD45^+CD64^+$ in the colon lamina propria and mesenteric lymph node or as $CD45^+F4/80^+$ in liver and epididymal white adipose tissue. Dendritic cells were defined as $CD45^+CD64^-CD11c^+MHCII^+$. (**D**) Quantification of frequency and geometric-mean fluorescence intensity of β7 integrin expression on pre-gated monocytes, macrophages, and dendritic cells from colonic lamina propria, mesenteric lymph nodes, and (**E**) liver and epididymal white adipose tissue from chow diet (CD) fed RAG-2-deficient mice (n = 9). Each dot represents data from one mouse. Values are represented as mean ± SD.

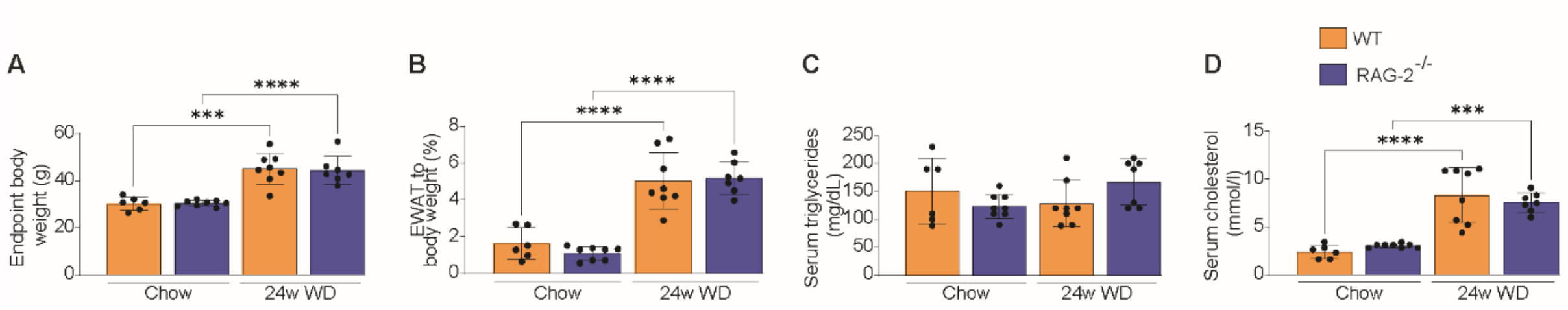

Figure S2. Metabolic syndrome can develop without lymphocytes. Wild type (WT) mice (shown in orange) and recombination activating gene-2-deficient (RAG-$2^{-/-}$) mice (shown in blue) were fed for 24 weeks with chow diet (CD, WT (n = 6), Rag-$2^{-/-}$ (n = 8)) or Western diet (WD, WT (n = 8), RAG-$2^{-/-}$ (n = 7)). (**A**) Endpoint body weight, (**B**) epididymal white adipose tissue (EWAT)/body weight ratio, (**C**) serum triglycerides, and (**D**) serum cholesterol. Values are represented as mean ± SD. Outliers were identified by ROUT method and statistical significance was calculated by two-way ANOVA and Tukey post-test. ***$p \leq 0.001$, **** $p \leq 0.0001$.

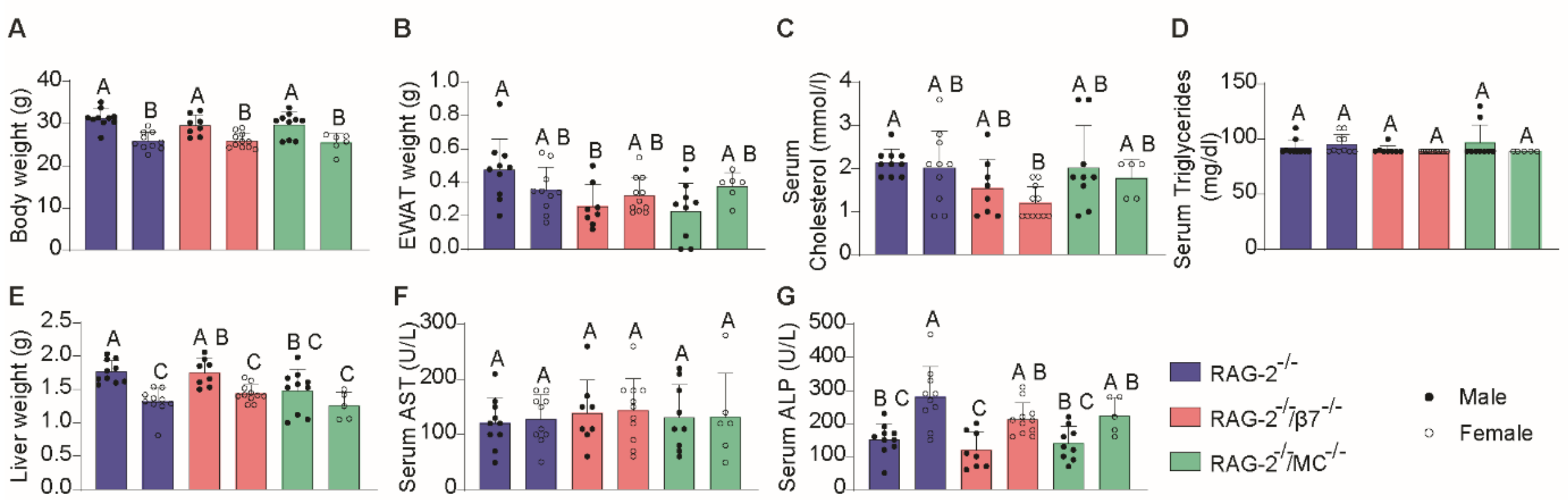

Figure S3. Loss of β7 integrin or MAdCAM-1 has no general impact on the health status of recombination activating gene-2-deficient (RAG-$2^{-/-}$) mice. The strains analyzed were RAG-$2^{-/-}$ mice (shown in blue, male (n = 10), female (n = 10)), RAG-2/β7 integrin double-deficient (RAG-$2^{-/-}$/β$7^{-/-}$) mice (shown in red, male (n = 8), female (n = 11)), and RAG-2/MAdCAM-1 double-deficient (RAG-$2^{-/-}$/MAdCAM-$1^{-/-}$) mice (shown in green, male (n = 10), female (n = 6)). These mice were at least 52 weeks of age. (**A**) Endpoint body weight, (**B**) epididymal white adipose tissue (EWAT) weight, (**C**) serum cholesterol, (**D**) serum triglycerides, (**E**) liver weight, (**F**) serum aspartate transaminase (AST), and (**G**)

serum alkaline phosphatase. Outliers were detected by ROUT method and statistical significance was calculated by the one-way ANOVA (**A** - **G**). Values are represented as mean ± SD (**A** - **G**). Results of pairwise comparisons between groups are shown as letters above the bars. Two groups that are named with the same letter show no statistical difference between each other while two groups with different letters are statistically significant with a p value smaller than 0.05.

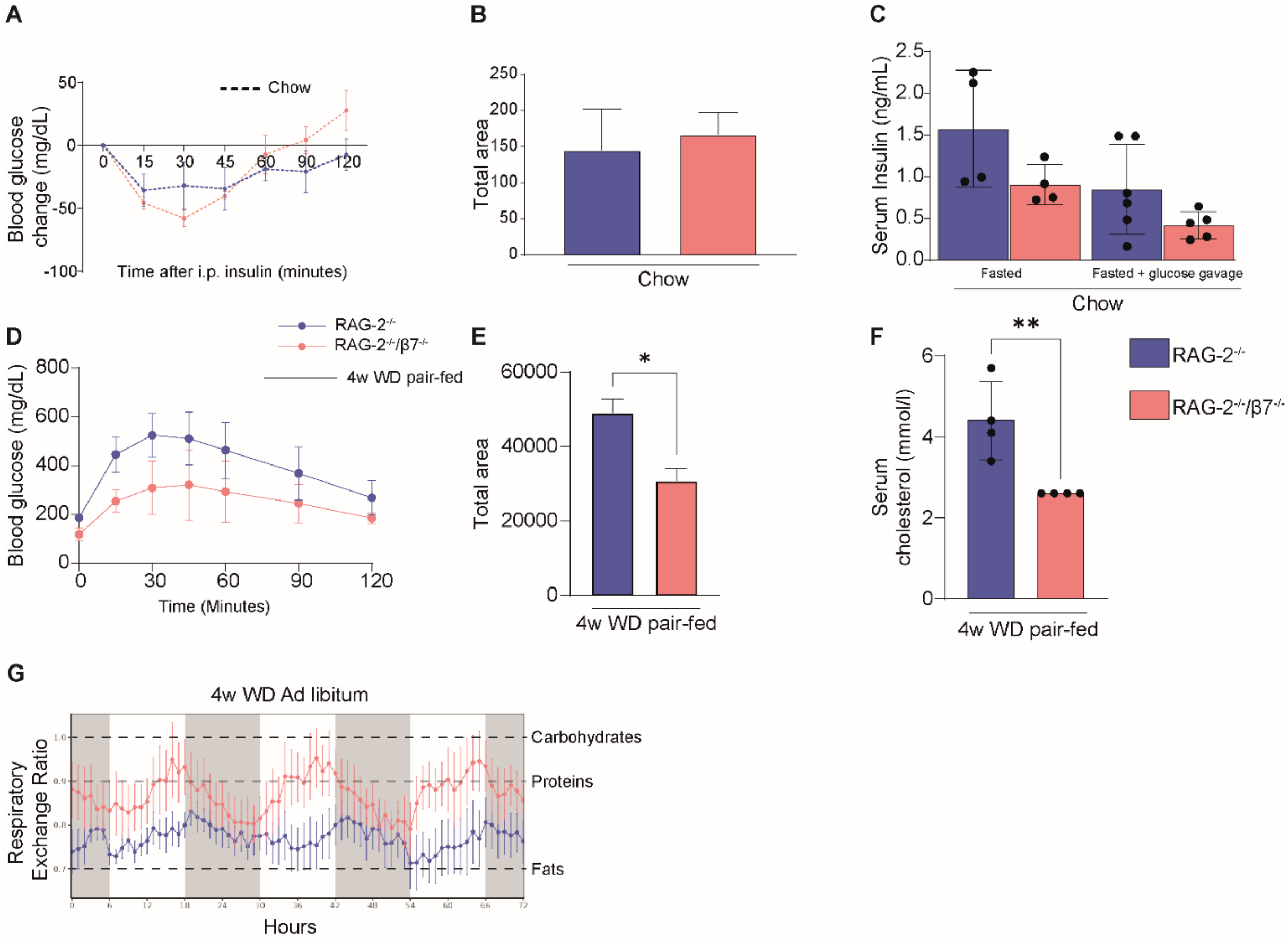

Figure S4. β7-integrin deficiency improves insulin sensitivity, protects from glucose intolerance and hypercholesteremia despite reduced food supply, and alters the respiratory exchange ratio (RER) in ad libitum WD-fed RAG-2-deficient mice. (**A – C**) Insulin sensitivity was compared between RAG-2-deficient (RAG-$2^{-/-}$) mice (blue, n = 5) and RAG-2/β7-integrin double-deficient (RAG-$2^{-/-}$/β$7^{-/-}$) mice (red, n = 4) under chow-fed conditions. Shown are (**A**) the endpoint insulin tolerance tests and (**B**) the corresponding area under the curve (AUC). (**C**) Serum insulin levels were measured in chow-fed RAG-$2^{-/-}$ mice (blue, n = 4–6) and RAG-$2^{-/-}$/β$7^{-/-}$ mice (red, n = 4–5) after 6 hours of fasting and following oral glucose administration (2 g/kg) after fasting. (**D – F**) RAG-$2^{-/-}$ mice (shown in blue, n = 4), and RAG-$2^{-/-}$/β$7^{-/-}$ mice (shown in red, n = 4) were compared in a pair-feeding experiment where they received for 4 weeks daily 3 g of Western diet (WD). Shown are the endpoint values of (**D**) glucose tolerance tests, and (**E**) corresponding quantification as AUC, and (**F**) serum cholesterol levels. (**G**) Metabolic cage studies were performed on 4 week ad libitum WD-treated RAG-$2^{-/-}$ mice (shown in blue, n = 4), and RAG-$2^{-/-}$/β$7^{-/-}$ mice (shown in red, n = 4) and RER was calculated as the ratio of the volume of carbon dioxide exhaled to volume of oxygen inhaled (VCO_2/VO_2). Statistical significance was calculated by unpaired t-test (**B – C**, **E** – **F**). Values are represented as mean ± SD (**A, C – D, F – G**) or mean ± SEM (**B**, **E**). * $p \leq 0.05$.

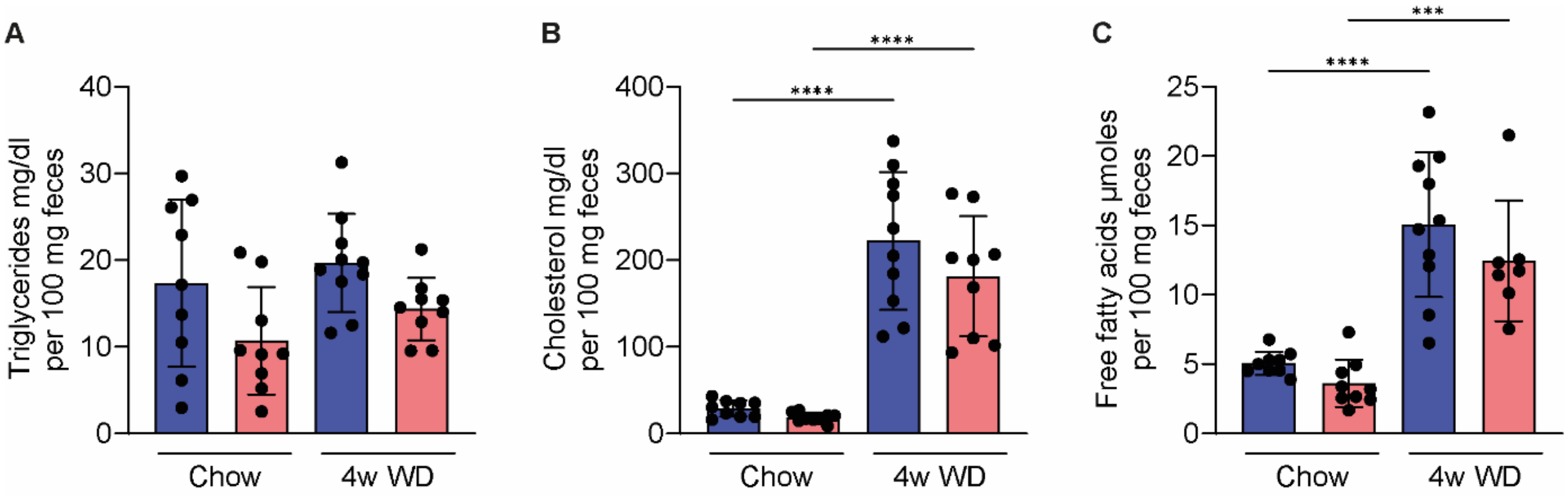

Figure S5. β7-integrin deficiency does not affect fecal lipid excretion in RAG-2-deficient mice. (**A-C**) Fecal samples were collected at baseline (chow) and after 4 weeks of Western diet (WD) treatment from RAG-2$^{-/-}$ mice (chow, n = 9; 4 weeks WD, n = 10) and RAG-2$^{-/-}$/β7$^{-/-}$ mice (chow, n = 9; 4 weeks WD, n = 7-9). Fecal lipids were extracted from dried fecal material by homogenization in chloroform:methanol (2:1, v/v), followed by phase separation with water. Extracted lipids were analyzed for (**A**) triglycerides, (**B**) cholesterol, and (**C**) free fatty acids, and values were normalized to 100 mg of starting fecal material. Statistical significance was determined by two-way ANOVA (**A-C**). Data are presented as mean ± SD. ***p ≤ 0.001, ****p ≤ 0.0001.

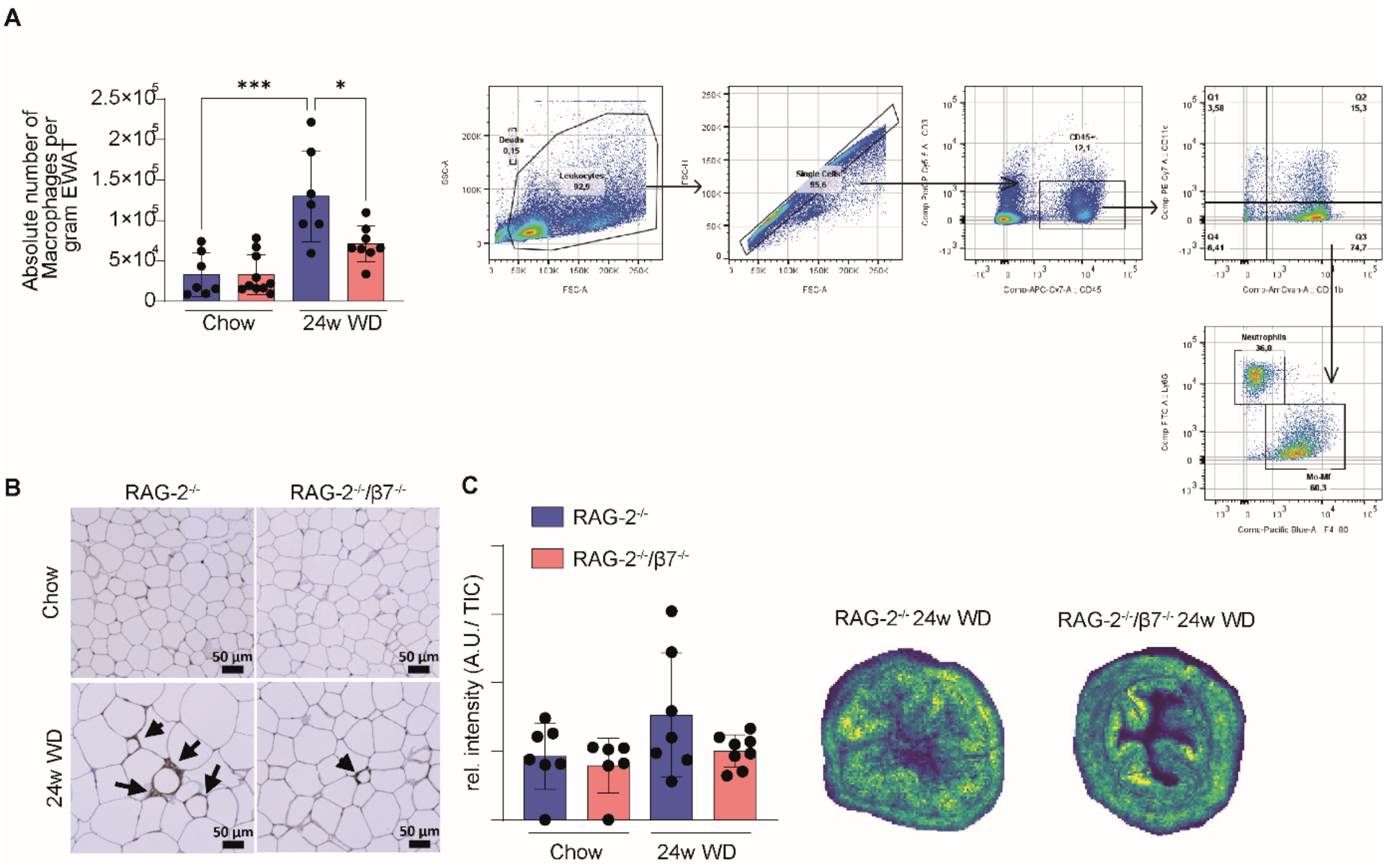

Figure S6. RAG-2-deficient (RAG-2$^{-/-}$) mice (shown in blue) and RAG-2/β7 integrin double-deficient (RAG-2$^{-/-}$/β7$^{-/-}$) mice (shown in red) were fed for 24 weeks with chow diet (CD) or Western diet (WD). (**A-C**) Quantification of macrophages after 24 weeks of CD or WD treatment in RAG-2$^{-/-}$ (CD-fed, (n = 7), WD-fed, (n = 7)) and RAG-2$^{-/-}$/β7$^{-/-}$ (CD-fed, (n = 6-9), WD-fed, (n = 8)) mice. (**A**) Flow cytometric analysis of macrophages (CD45^{+} CD11b^{+} F4/80^{+}) in epididymal white adipose tissue (EWAT) with

corresponding gating strategy, and (**B**) representative images of CD68-stained EWAT sections (original magnification x 20, scale bar = 50 µm). Areas with CD68⁺ crown-like structures are labeled with black arrows. (**C**) Quantification and representative images of colonic Fcgr1 measured by matrix-assisted laser desorption ionization mass spectrometric imaging. Statistical significance was calculated by two-way ANOVA test (**A**) or t-test (**C**). Values are represented as mean ± SD. $^{*}p \leq 0.05$, $^{**} p \leq 0.01$, $^{***} p \leq 0.001$.

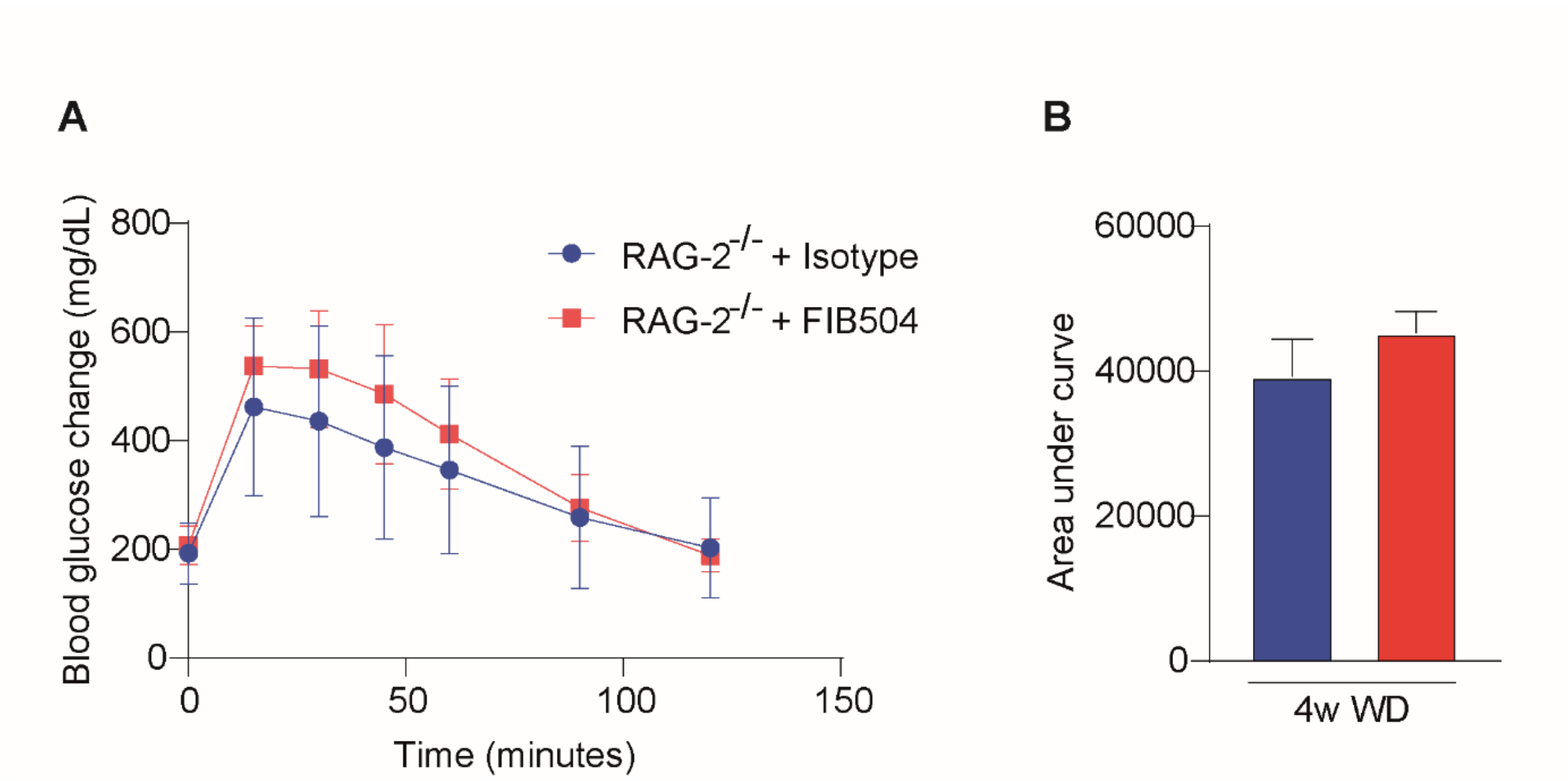

Figure S7. RAG-2$^{-/-}$ mice were fed for 4 weeks with a Western diet (WD) and given an IgG isotype antibody (shown in blue, (n =9)) or FIB 504 monoclonal antibody against β7 integrin (shown in red, (n = 9)). (**A**) Endpoint insulin tolerance test, and (**B**) corresponding quantification as area under curve. Statistical significance was calculated by t-test and values are represented as mean ± SD (**A**) and mean ± SEM (**B**).

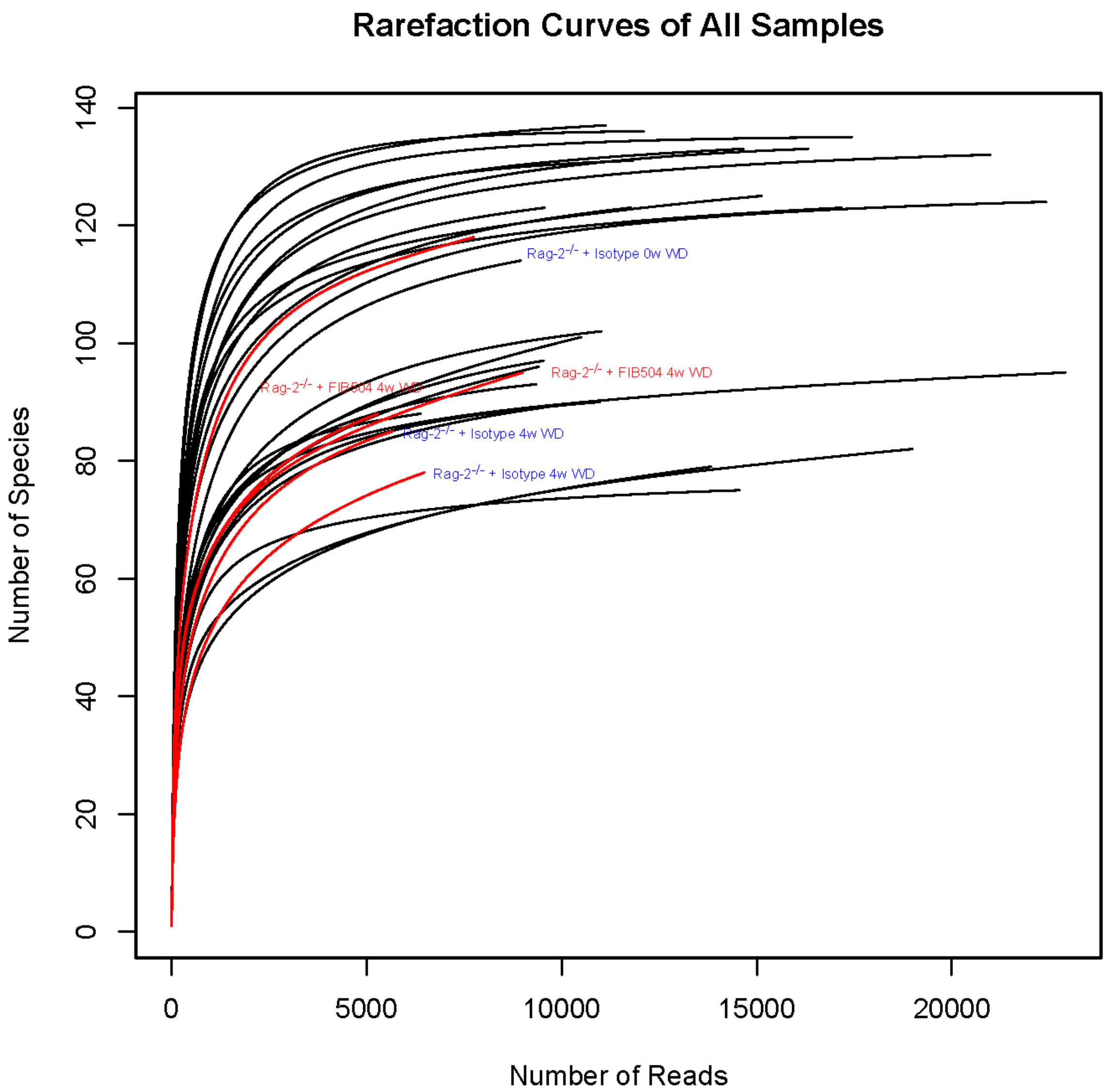

Figure S8. Rarefaction curves depicting sequencing depth. The number of observed species in each sample was plotted against the number of reads acquired, to ensure sufficient sequencing depth. The curve was generated in R using Rhea [1].

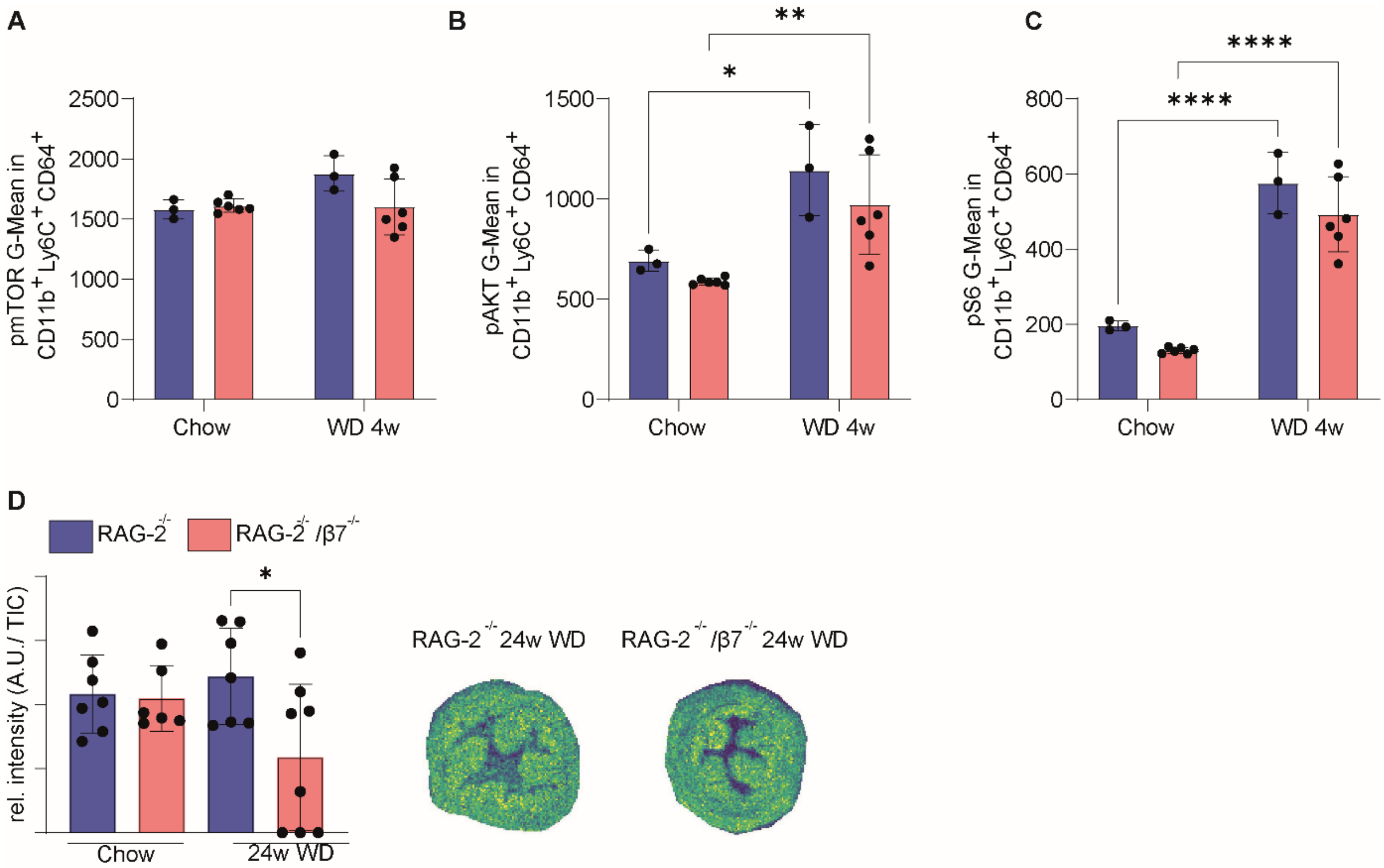

Figure S9. (**A-C**) Analysis of mTOR/Akt signaling pathway activation and ribosomal protein S6 in monocytes-macrophages (CD11b$^+$CD64$^+$Ly6C$^+$) from the small intestinal lamina propria of 4-week WD or chow diet (CD) treated RAG-2$^{-/-}$ (CD-fed: n = 3, WD-fed: n = 6) and RAG-2$^{-/-}$/β7$^{-/-}$ (CD-fed: n = 6, WD-fed: n = 9) mice by flow cytometry. Data are shown as Geometric mean fluorescence intensity (G-Mean) of (**A**) phosphorylated mechanistic Target of Rapamycin (mTOR), (**B**) protein kinase B (AKT), and (**C**) ribosomal protein S6 (S6). Statistical significance was calculated by two-way ANOVA test (A-C) and Tukey's post-test. *$p \leq 0.05$, ** $p \leq 0.01$, **** $p \leq 0.0001$. (**D**) Quantification and representative images of phosphorylated Akt1 measured by matrix-assisted laser desorption ionization mass spectrometric imaging in the colon of RAG-2$^{-/-}$ (CD-fed: n = 7, WD-fed: n = 7) and RAG-2$^{-/-}$/β7$^{-/-}$ (CD-fed: n = 6, WD-fed: n = 8) mice after 24 weeks of feeding. Statistical significance was calculated by one-way ANOVA test. *$p \leq 0.05$.

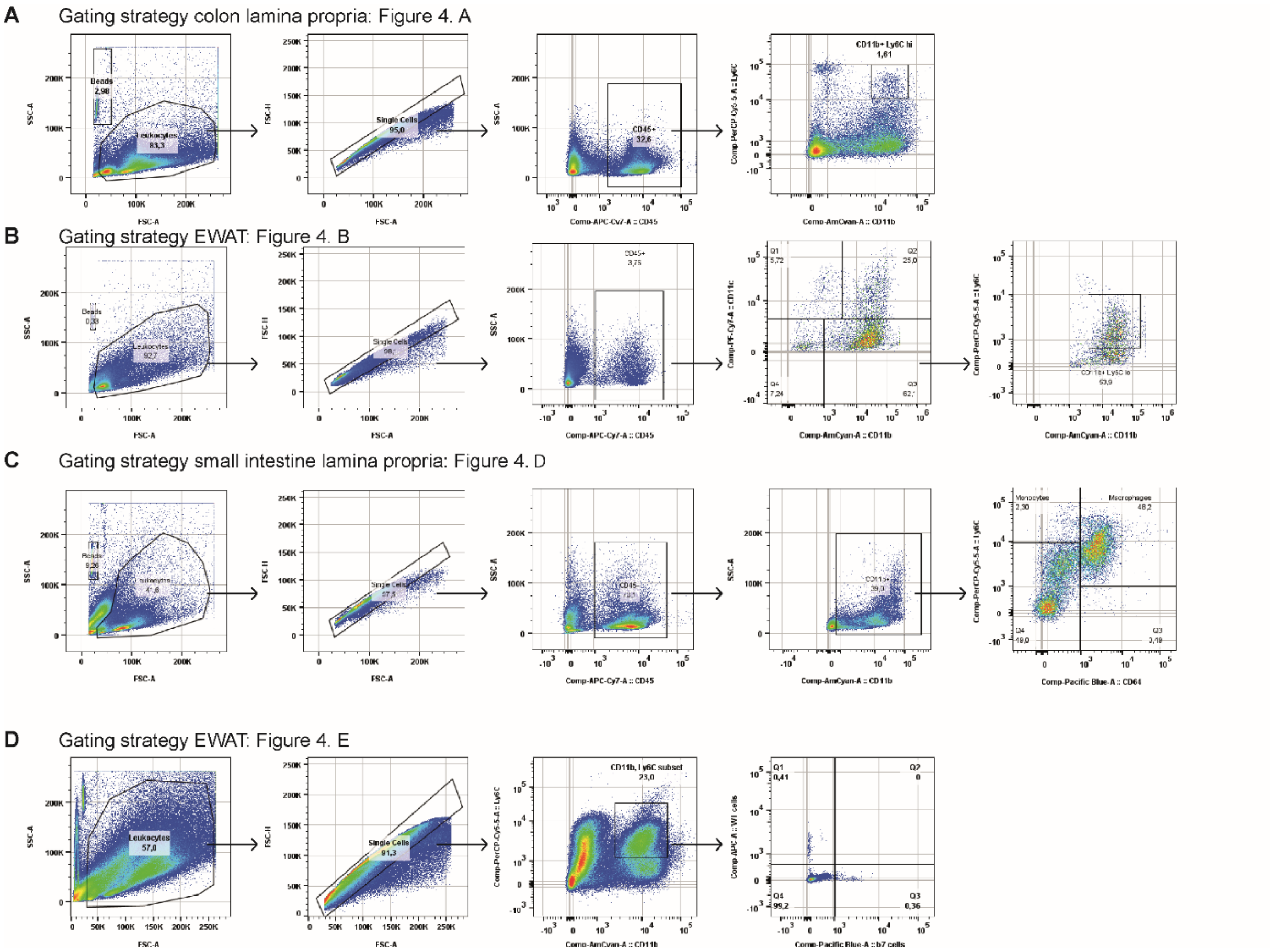

Figure S10. Representative immune cell subset gating strategies by multiparameter flow cytometry for monocytes and macrophages in RAG-2-deficient mice (**corresponding to Figure 4 in main text**). All samples were first gated as leukocytes on FSC-A against SSC-A followed by exclusion of doublet cells by gating on FSC-A against FSC-H. **(A)** Inflammatory monocytes ($CD45^+$ $CD11b^+$ $Ly6C^{hi}$) in colonic lamina propria. **(B)** Inflammatory monocytes ($CD45^+$ $CD11b^+$ $Ly6C^+$) in epididymal white adipose tissue (EWAT). **(C)** Macrophages ($CD45^+$ $CD11b^+$ $CD64^+$) in small intestinal lamina propria. **(D)** proportions of immigrated monocytes ($CD11b^+$ $Ly6C^+$) in EWAT.

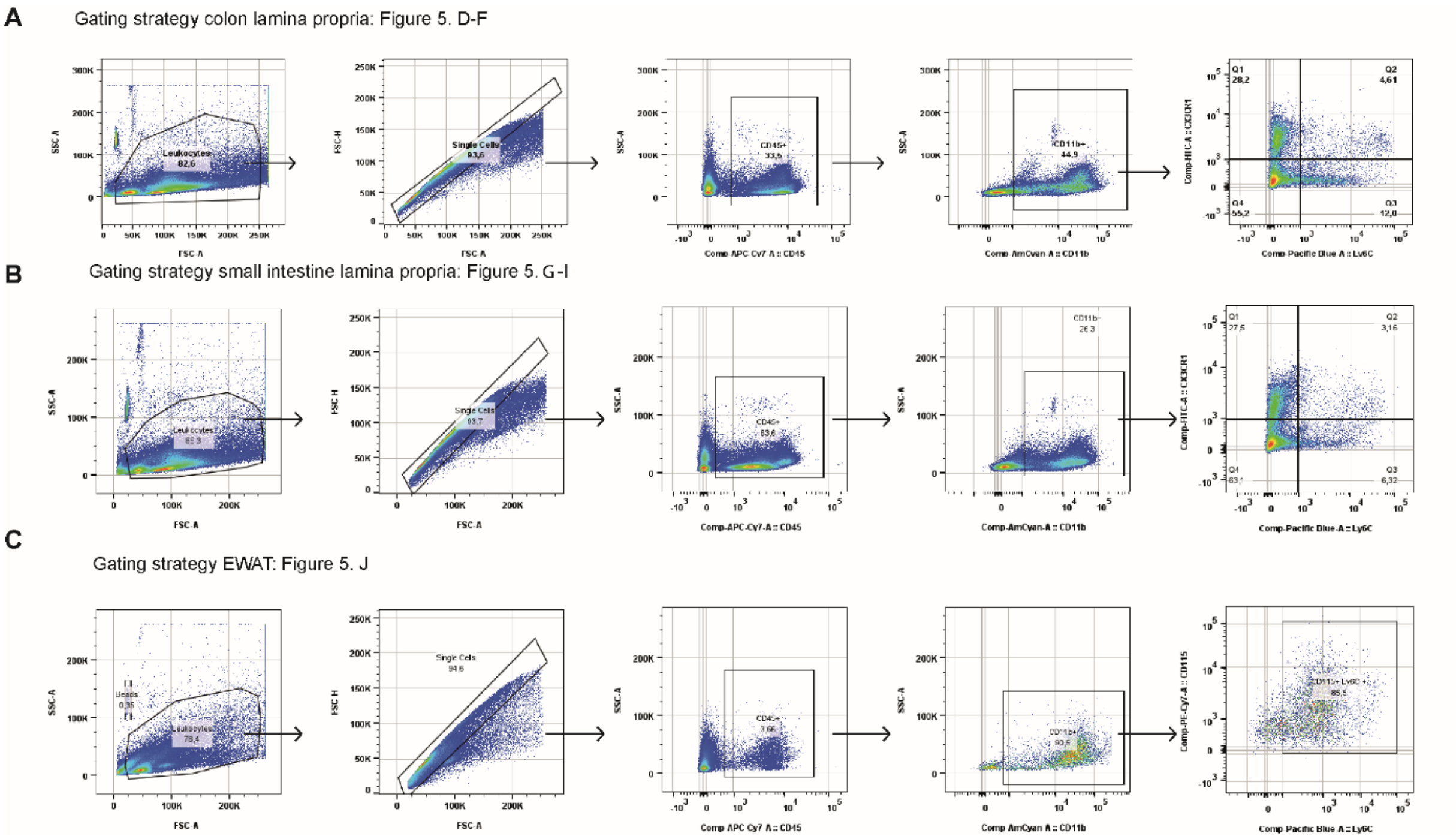

Figure S11. Representative immune cell subset gating strategies by multiparameter flow cytometry for monocytes and macrophages in RAG-2-deficient mice treated with monoclonal antibodies against β7 Integrin or IgG isotype (**corresponding to Figure 5 in main text**). All samples were first gated as leukocytes on FSC-A against SSC-A followed by exclusion of doublet cells by gating on FSC-A against FSC-H. **(A)** Colonic monocyte populations ($Ly6C^{+}CX3CR1^{-}$, $Ly6C^{+}CX3CR1^{+}$, and $Ly6C^{-}CX3CR1^{+}$) represented as frequency of $CD45^{+}$ cells, **(B)** small intestinal monocyte populations ($Ly6C^{+}CX3CR1^{-}$, $Ly6C^{+}CX3CR1^{+}$, and $Ly6C^{-}CX3CR1^{+}$) represented as frequency of $CD45^{+}$ cells, and (**C**) $Ly6C^{+}CD115^{+}$ monocyte frequency of $CD11b^{+}$ myeloid cells in epididymal white adipose tissue (EWAT).

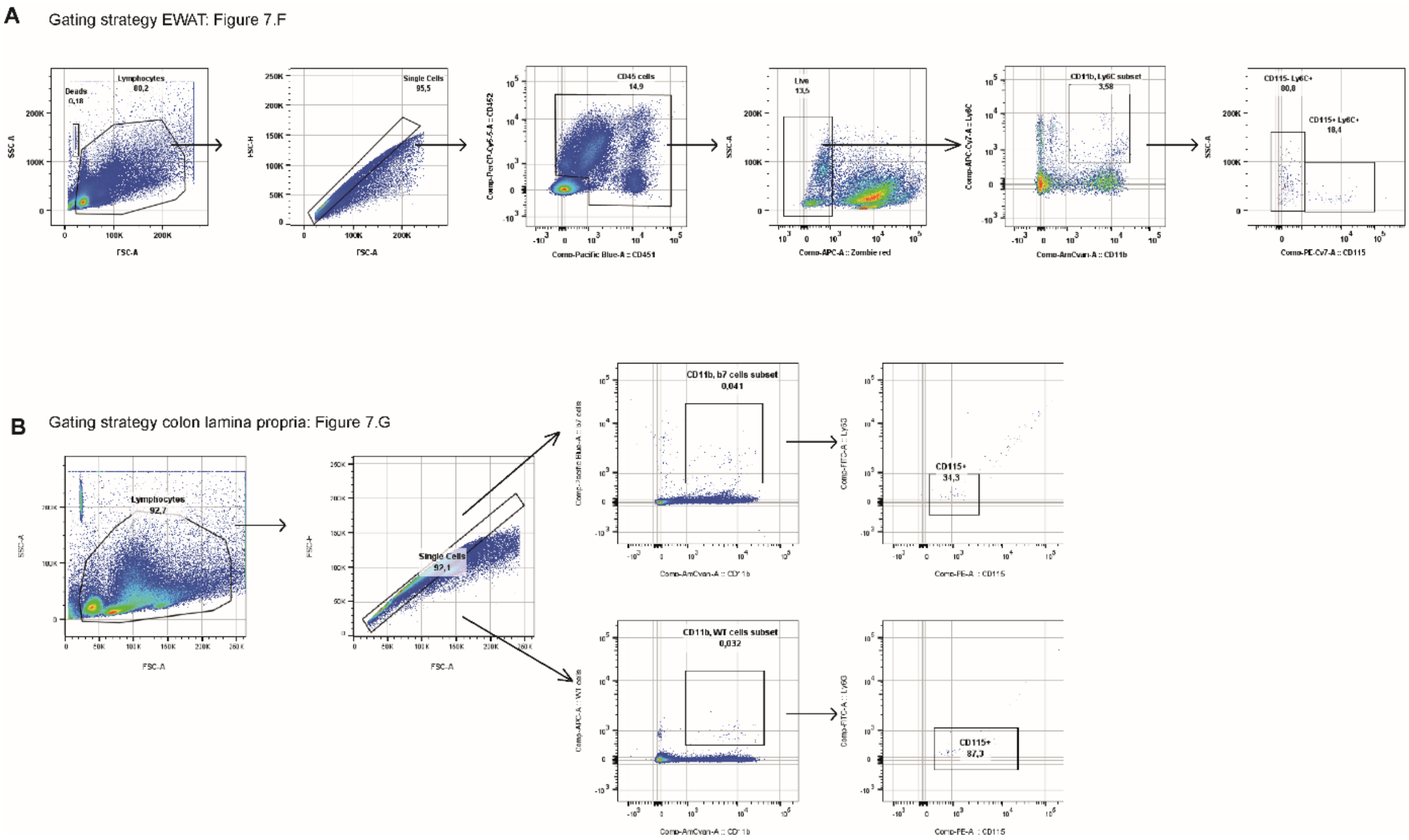

Figure S12. Representative immune cell subset gating strategies by multiparameter flow cytometry for monocytes in CCR-2-deficient mice (**corresponding to Figure 7 in main text**). All samples were first gated as leukocytes on FSC-A against SSC-A followed by exclusion of doublet cells by gating on FSC-A against FSC-H. **(A)** Monocytes ($CD45^+$ Live $CD11b^+$ $Ly6C^+$ $CD115^+$) in epididymal white adipose tissue (EWAT). **(B)** proportions of immigrated monocytes ($CD11b^+$ $CD115^+$ $Ly6G^-$) in EWAT.

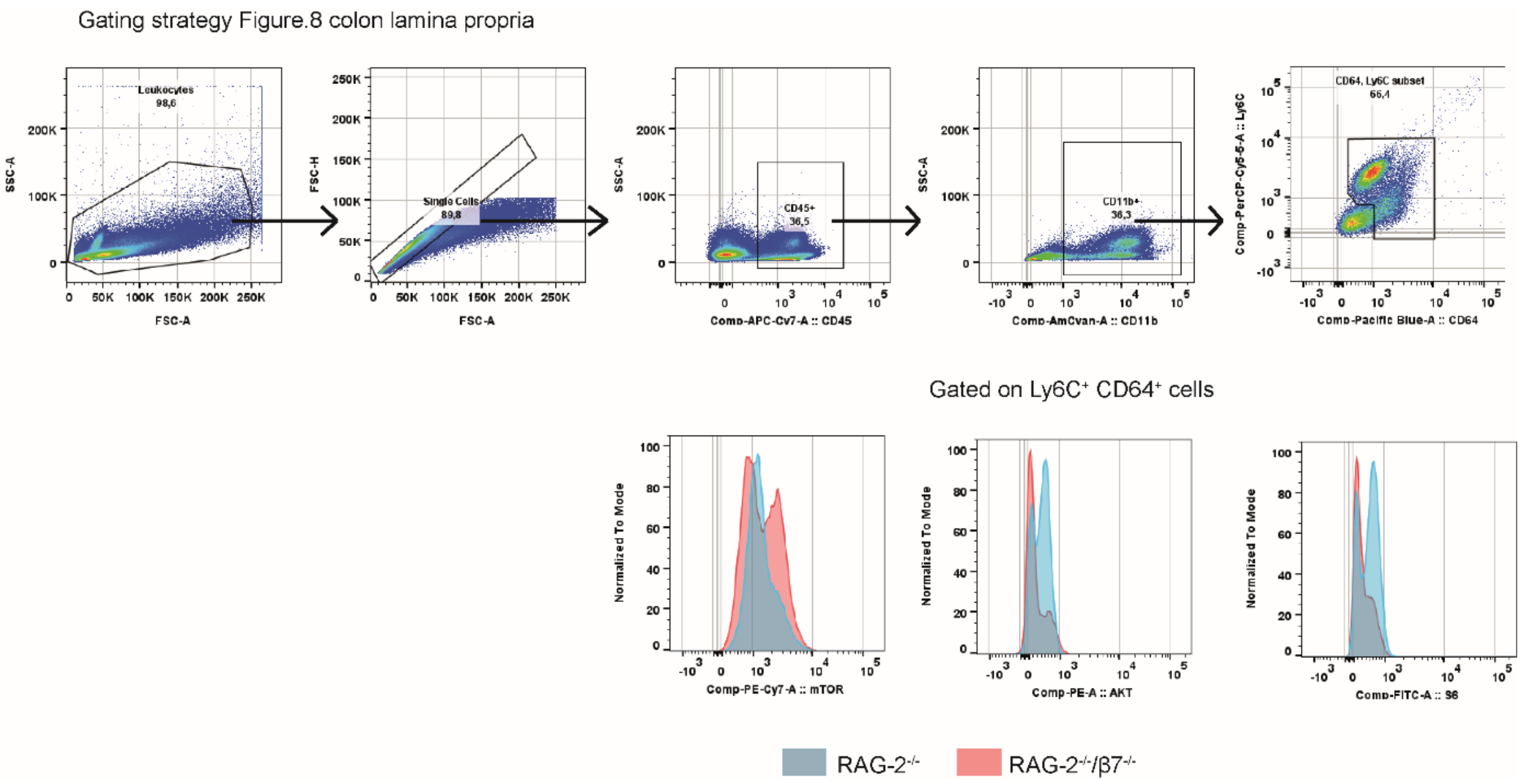

Figure S13. Immune cell subset gating strategies by multiparameter flow cytometry for levels of phosphorylated mTOR, AKT and S6 on monocytes and macrophages in RAG-2-deficient mice (**corresponding to Figure 8 in main text**). All samples were first gated as leukocytes on FSC-A against

SSC-A followed by exclusion of doublet cells by gating on FSC-A against FSC-H. Monocytes-macrophages from colon and small intestine lamina propria were defined as $CD45^+$ $CD11b^+$ $CD64^+$ $Ly6C^+$. Levels of phosphorylated mTOR, AKT and S6 were measured as difference in geometric mean of fluorescence intensity between antibody stained and unstained samples of the same genotype.

References

1. Lagkouvardos, I., et al., *Rhea: a transparent and modular R pipeline for microbial profiling based on 16S rRNA gene amplicons.* PeerJ, 2017. **5**: p. e2836.
